# Classical/Non‐classical Polyoxometalate Hybrids

**DOI:** 10.1002/chem.201604238

**Published:** 2016-10-05

**Authors:** Natalya V. Izarova, Beatrix Santiago‐Schübel, Sabine Willbold, Volkmar Heß, Paul Kögerler

**Affiliations:** ^1^ Jülich-Aachen Research Alliance (JARA-FIT) and Peter Grünberg Institute 6Forschungszentrum Jülich52425JülichGermany; ^2^ Central Institute for Engineering, Electronics and Analytics 3Forschungszentrum Jülich52425JülichGermany; ^3^ Institute of Inorganic ChemistryRWTH Aachen University52074AachenGermany

**Keywords:** ^77^Se NMR, ESI mass spectrometry, palladium, polyoxometalates, tungsten

## Abstract

Two polyanions [Se^I V^_2_Pd^II^_4_W^VI^_14_O_56_H]^11−^ and [Se^I V^_4_Pd^II^_4_W^VI^_28_O_108_H_12_]^12−^ are the first hybrid polyoxometalates in which classical (Group 5/6 metal based) and non‐classical (late transition‐metal based) polyoxometalate units are joined. Requiring no supporting groups, this co‐condensation of polyoxotungstate and isopolyoxopalladate constituents also provides a logical link between POM‐Pd^II^ coordination complexes and the young subclass of polyoxopalladates. Solid‐state, solution, and gas‐phase studies suggest interesting specific reactivities for these hybrids and point to several potential derivatives and functionalization strategies.

The chemistry of palladium‐containing polyoxometalates (POMs) has experienced impressive development over the past decade,^1^ with progress primarily concentrated on two areas. The first is defined by conventional Pd^II^ coordination complexes of lacunary polyoxotungstates (POTs), [Pd^II^_*n*_(X_*m*_W_*p*_O_*q*_)_r_]^*Z*−^, where Pd^II^ ions in square‐planar environments coordinate oxygen atoms of vacant sites of POT ligands, resulting in a diverse range of structures incorporating one to four Pd^II^ centers.^2^ Such species are convenient precursors for highly stable suspensions of POT‐stabilized Pd^0^ nanoparticles, which can be obtained at mild conditions in aqueous media.^3^ Some of the Pd‐POT complexes were also shown to act as pre‐catalysts for various organic transformations.2m, 4 In these complexes, the Pd^II^ centers typically lack a direct connection, with the only exception in [Pd^II^_4_(*α*‐P_2_W_15_O_56_)_2_]^16−^, where two out of four Pd^II^ ions are bridged via O atoms of two phosphate groups.2p

In the second main area, formed by so‐called polyoxopalladates (POPds), the Pd^II^ centers, in contrast, act as addenda ions themselves. Here, the elementary PdO_4_ building blocks are condensed via corners and edges, typically also involving external RXO_3_^*z*−^ heterogroups stabilizing the discrete {Pd_*x*_O_*y*_} entity.^5^, ^6^ About 50 of these non‐classical POMs are known today, incorporating up to 84 Pd^II^ ions. One of the most stable POPds archetype comprises species of general composition [MPd^II^_12_O_8_(RXO_3_)_8_]^*z*−^ ({MPd_12_}), where a heterometal ion M^*z*′+^ in a cubic O_8_ environment is encapsulated in the cuboid‐shaped {Pd^II^_12_O_8_(RXO_3_)_8_} shell (RX=Se^IV^, OAs^V^, PhAs^V^, OP^V^, PhP^V^).^6^

Recently, Cronin and co‐workers also reported several polyanions that can be considered as complexes of seleno‐ and tellurotungstates {X_*n*_W_*m*_O_*p*_} with selenite‐ or tellurate‐supported multinuclear Pd^II^‐based fragments.^7^ In two isomeric [H_*x*_Pd^II^_10_Se^I V^_10_W_52_O_206_]^(40−*x*)−^ polyanions, two {Pd_5_Se_2_O_2_} units are coordinated to {*B*‐α‐SeW_9_O_33_} and {γ‐Se_2_W_14_O_56_} POT moieties. In [Pd^II^_6_Te^I V^_19_W_42_O_190_]^40−^ two identical {Pd_3_Te_3_O_3_} groups are stabilized by six {α‐TeW_7_O_27_} lacunary POTs.^7^

Yet, up to now there was no systematic investigation on how to achieve commensurate reaction conditions that allow to co‐condense, and thus cleanly interface, classical POTs and non‐classical POPds. We thus explored the possibility to prepare hybrid polyoxopalladatotungstates [X_*n*_Pd^II^_*m*_W^IV^_*p*_O_*q*_]^*z*−^, where both Pd^II^ and W^VI^ centers act as addenda centers of their individual POM units, without the need for any additional external stabilizing groups. Herein we report two first examples of such hybrid palladatotungstates, [Se^I V^_2_Pd^II^_4_W^VI^_14_O_56_H]^11−^ (**1**) and [Se^I V^_4_Pd^II^_4_W^VI^_28_O_108_H_12_]^12−^ (**2**), crystallized as hydrated mixed cesium/sodium salts Cs_4_Na_3_H_4_[Se_2_Pd_4_W_14_O_56_H]⋅ 18 H_2_O⋅0.3 CsOAc⋅0.2 NaOAc (**CsNa‐1**; OAc^−^=acetate) and Cs_9.5_Na_2.5_[Se_4_Pd_4_W_28_O_108_H_12_]⋅30 H_2_O (**CsNa‐2**), respectively, and their characterization in the solid state, aqueous solutions, and gas phase.

The polyanions **1** and **2** have been prepared in reactions of [Se^I V^_6_W^VI^_39_O_141_(H_2_O)_3_]^24−^ ({Se_6_W_39_})^8^ with Pd^II^ nitrate in different aqueous media (Supporting Information, Scheme S1). The {Se_6_W_39_} precursor possesses a cyclic structure, where three {γ‐Se_2_W_12_O_46_} units are alternating with three *trans*‐{O=W(H_2_O)} groups. In aqueous solution it slowly decomposes, releasing {Se_*x*_W_*y*_O_*z*_} fragments^8^ and thus could act as a source for preparation of diverse tungstoselenite complexes.^9^ The Cs^+^ counterions seem to play an important role for isolation of **1** and **2** as pure crystalline materials owing to relatively low solubility of the hydrated Cs^+^ salts. Alternatively, a Rb^+^/Na^+^ salt of **1** can be successfully prepared by replacing CsNO_3_ with RbNO_3_ in the synthesis of **CsNa‐1**. With no additional counterions only the hydrated sodium salt of paratungstate‐*B* ([H_2_W_12_O_42_]^10−^) could be isolated from the reaction medium for preparation of **1** as a crystalline product. The paratungstate‐*B* salt is also sometimes present as an impurity to **CsNa‐1**, which could be purified in this case by recrystallization from 0.25 m NaOAc aqueous solution (pH 6.7). Similar recrystallization of **CsNa‐2** leads to formation of a mixture of **CsNa‐1**, **CsNa‐2**, and other undefined products. The purity and composition of the compounds was further confirmed by elemental analysis, PXRD, TGA, and XPS (see the Supporting Information for details).

**CsNa‐1** crystallizes in the orthorhombic space group *Pnnm*. The polyanion **1** exhibits idealized *C*_2*v*_ symmetry and comprises an [α‐Se_2_W_14_O_52_]^12−^ POT moiety ({α‐Se_2_W_14_}) supporting a {Pd_4_O_4_} fragment (Figure 1).

**Figure 1:**
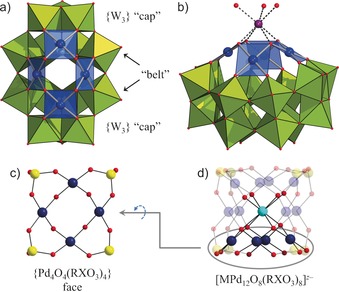
Structure of **1** (a) and the {(H_2_O)_3_Na}‐**1** associate (b); comparison with the {Pd_4_O_4_(RXO_3_)_4_} fragment (c) in the cuboid‐shaped polyoxopalladate [MPd_12_O_8_(RXO_3_)_8_]^*z*−^ (d). WO_6_ lime green, PdO_4_ blue polyhedra; Pd blue, Se/X yellow, O red, Na purple, M light blue. The R groups in {MPd_12_} are omitted for clarity.

The {α‐Se_2_W_14_} unit can be compared to a hypothetical tetralacunary Wells–Dawson‐type {α‐P_2_W_14_O_54_} fragment (Supporting Information, Figure S3), with two neighboring {W_2_O_10_} groups, composed of two edge‐shared {WO_6_} octahedra, removed from the inner {W_6_} belts of [α‐P_2_W_18_O_62_]^6−^ ({α‐P_2_W_18_}; Supporting Information, Figure S3a/b), each one from one belt. The Se^IV^ ions in {α‐Se_2_W_14_} adopt a trigonal pyramidal environment with the outwards oriented lone pair (Supporting Information, Figure S3d; Se−O 1.677(16)–1.725(15) Å). The formation of {α‐Se_2_W_14_} from the {γ‐Se_2_W_12_} building blocks of the {Se_6_W_39_} precursor requires attachment of two additional W^VI^ ions to {γ‐Se_2_W_12_}, each of which is completing the outer {W_3_} cap of the POT fragment, combined with {γ‐Se_2_W_14_} isomerization by rotation of both {W_3_} caps by 60° (Supporting Information, Figure S3). The same {α‐Se_2_W_14_} building blocks have been recently observed in [Fe_6_Se_6_W_34_O_124_(OH)_16_]^18−^ polyanions.^9^ At the same time, the arrangement of W^VI^ centers in {α‐Se_2_W_14_} is different from that in the actual {α‐P_2_W_14_O_54_} moieties that, for example, form [H_12_Fe_8_P_4_W_28_O_120_]^16−^ and [(W_4_Mn_4_O_12_)(P_2_W_14_O_54_)_2_]^20−^complexes.^10^ In fact, these {α‐P_2_W_14_O_54_} building blocks are the structural isomers to the hypothetical {α‐P_2_W_14_} units discussed above, and can be obtained from {α‐P_2_W_18_} polyanions by removing not {W_2_O_10_} but rather corner‐sharing {W_2_O_11_} units from its inner belts (Supporting Information, Figure S3c). It is also different in {γ‐Se_2_W_14_} moieties constructing the reported [H_*x*_Pd_10_Se_10_W_52_O_206_]^*n*−[7]^ (see above) and [Fe_10_Se_8_W_62_O_222_(OH)_18_(H_2_O)_4_]^28−[9]^ complexes where the two {W_3_} caps are rotated by 60° relative to their orientation in the α isomer (Supporting Information, Figure S3e).

The four Pd^II^ centers in the {Pd_4_O_4_} fragment form a rectangle (Pd⋅⋅⋅Pd 3.360(2)–3.375(2) Å) and are linked by four μ_2_‐O sites (Figure 1 a). All square‐planar Pd^II^O_4_ (Pd−O 1.976(14)‐2.010(15) Å) include two *cis*‐positioned μ_2_‐O of the {Pd_4_O_4_} fragment as well as two O_POT_ atoms: two Pd^II^ centers bind to the {W_3_} caps and two to the belts of {α‐Se_2_W_14_} (Figure 1 a). Based on bond valence sums, the proton in **1** is disordered over the four μ_2_‐O atoms linking the Pd^II^ centers. These oxygens also coordinate to a {Na(OH_2_)_3_}^+^ counterion (Figure 1 b; Na−O 2.42(2)–2.53(2) Å).

The direct connection between the Pd^II^ centers by oxo ligands as well as the complete integration of the POPd {Pd_4_O_4_} moiety in the POM framework allow to consider **1** as a genuine hybrid polyoxopalladatotungstate. Interestingly, the structure of {Pd_4_O_4_} unit in **1** compares to the {Pd_4_O_4_(RXO_3_)_4_} face in the cuboid‐shaped {MPd_12_} POPds (Figure 1 c/d1), with the RXO_3_^*n*−^ groups stabilizing the {MPd_12_O_8_} core replaced by {α‐Se_2_W_14_}. Moreover, the Na^+^ attachment to {Pd_4_O_4_} in **1** is similar to the connection mode between the central M^*z*+^ ion and the {Pd_4_O_4_(RXO_3_)_4_} face in {MPd_12_} nanocubes (Figure 1 b/d1). This suggests that the {Pd_4_O_4_} group in **1** possesses reactivity towards oxophilic heterometals.

The total number of metal centers in **1** allows for an analogy between **1** and Wells–Dawson‐type polyanions {α‐P_2_W_18_}.^11^ Both POMs comprise two central heteroatoms surrounded by 18 addenda metal ions. However the {Pd_4_} rectangle in **1** is rotated by 45° in comparison to the {W^VI^_4_} rectangle in {α‐P_2_W_18_} if the latter is formally decomposed into the above‐mentioned hypothetical {α‐P_2_W_14_} fragment and four W^VI^ centers (Supporting Information, Figure S4), possibly enforced by the square‐planar Pd coordination mode in **1** relative to the octahedral W^VI^O_6_ groups. This analogy prompted us to probe the possibility to form lacunary derivatives of **1** at conditions similar to those for formation of {α_2_‐P_2_W_17_} and {α‐P_2_W_15_} from {α‐P_2_W_18_}. These experiments, however, only resulted in Cs_2_Na_3_[H_5_Pd_15_Se_10_O_10_(SeO_3_)_10_]⋅ca. 20 H_2_O⋅POPd,^12^ which suggests that decomposition of **1** proceeds first through release of Pd^II^ ions, followed by POT decomposition.

However the possibility of existence of unstable lacunary derivatives of {α/β/γ‐Se_2_Pd_4_W_14_} polyanions is evident from the structure of **2** obtained indirectly by reaction of {Se_6_W_39_} with Pd^II^ in water. The compound **CsNa‐2** crystallizes in the triclinic space group *P* $\bar{1}$ . The unit cell in **CsNa‐2** contains two identical polyanions **2**, each of which can be imagined as a dimer of two γ‐{(H_2_O)(OH)_2_Pd^II^_2_Se^I V^_2_W_13_O_49_} ({γ‐Pd_2_Se_2_W_13_}) units connected by two *trans*‐{O=W(H_2_O)} groups (Figure 2). In line with the previous discussion, the {γ‐Se_2_W_13_} structure can be understood as a {γ‐Se_2_W_12_} unit, present in {Se_6_W_39_}, binding a W^VI^ to complete one of the {W_3_} caps or, alternatively, as {γ‐Se_2_W_14_} (Supporting Information, Figure S3e), missing one W^VI^ ion in its {W_3_} cap. The two Pd^II^ ions in {γ‐Pd_2_Se_2_W_13_} assume a square planar environment, each coordinating two *cis*‐positioned oxygens of {γ‐Se_2_W_13_}: one from the W^VI^ ion in the {W_3_} cap and one from the {W_4_} belt (Figure 2 a). Furthermore, the two Pd^II^ ions are μ_2_‐OH‐bridged. One of the Pd^II^ ions additionally coordinates a terminal H_2_O, and its μ_2_‐O (Pd, W) ion in the *trans*‐position to the aqua ligand is protonated (Figure 2 a/c2; Supporting Information, Table S4). The second Pd^II^ ion is bound to *trans*‐{O=W(H_2_O)} group through the μ_2_‐O (Figure 2 b).

**Figure 2:**
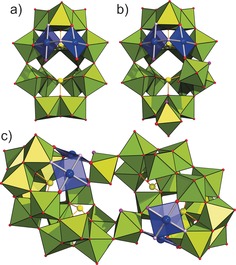
The structure of a {γ‐Pd_2_Se_2_W_13_} monomer (a) and a γ‐Pd_2_Se_2_W_13_{O=W(H_2_O)}_2_ moiety (b) in the polyanion **2** (c). WO_6_ lime green octahedra, PdO_4_ blue squares; Pd blue, Se yellow, O red spheres. The monoprotonated O atoms in the structure of **2** are highlighted in light purple, while aquo ligands are shown in pink.

Thus, the {γ‐Pd_2_Se_2_W_13_} fragment can be considered as a lacunary derivative of a hypothetical plenary {γ‐Pd_4_Se_2_W_14_} polyanion, lacking two Pd^II^ and one W^VI^ centers. It is interesting to note that the orientation of {Pd_4_O_4_} fragment in this {γ‐Pd_4_Se_2_W_14_} POM, in case it exists, would be similar to that in {α‐P_2_W_18_} and not in {α‐Pd_4_Se_2_W_14_}. Along with the μ_2_‐O ligand connecting it to Pd^II^ (see above), the W^VI^ center of each *trans*‐{O=W(H_2_O)} group also binds to an O atom of the neighboring {W_4_} belt of the same {γ‐Pd_2_Se_2_W_13_} monomeric unit as well as to the two O atoms of the incomplete {W_2_} cap group of the second {γ‐Pd_2_Se_2_W_13_} monomer, each of which belongs to different W^VI^ ions (Figure 2 b/c2). Interestingly, one of the H_2_O ligands of the *trans*‐{O=W(H_2_O)} groups is directed inward the polyanion, while the second one is pointed outward (Figure 2 c). Thus, considering the protonation sites, **2** is of *C*_1_ symmetry. Otherwise, it would possess a *C*_2_ axis passing through the center of a line connecting the W^VI^ centers of the two {O=W(H_2_O)} groups (Figure 2 c).

Owing to the presence of large Cs^+^ cations, the compounds **CsNa‐1** and **CsNa‐2** are only slightly soluble in water; however, their solubility is significantly increased in 0.25–0.5 m sodium and lithium acetate solutions (pH 6–7), especially upon heating to 65–70 °C. This allowed assessment of the solution behavior of **1** and **2** by ^77^Se NMR and UV/Vis spectroscopy (see the Supporting Information). Room‐temperature ^77^Se NMR of **1** in 0.25 m LiOAc solution (pH 6.2) exhibits a singlet at 1225.3 ppm (Figure 3), consistent with the presence of only one symmetrically non‐equivalent Se^IV^ ion in the crystal structure of **CsNa‐1** and with the observation of a singlet at 1202 ppm in the ^77^Se MAS NMR for this compound (Supporting Information, Figure S12). This indicates stability of **1** in aqueous medium in saturated solutions. The observed chemical shift is commensurate with those of Zn^II^ (1222.5 ppm) and Lu^III^ (1223.8 ppm)‐centered cuboid {MPd_12_Se_8_} POPds6c and is significantly upfield‐shifted compared to an aqueous SeO_2_ solution (pH 6.4; 1316.3 ppm). For comparison with other tungstoselenites, the {Se_6_W_39_} precursor (unstable in solution) gives a broad peak centered at 1289.1 ppm in ^77^Se MAS NMR.8a

**Figure 3:**
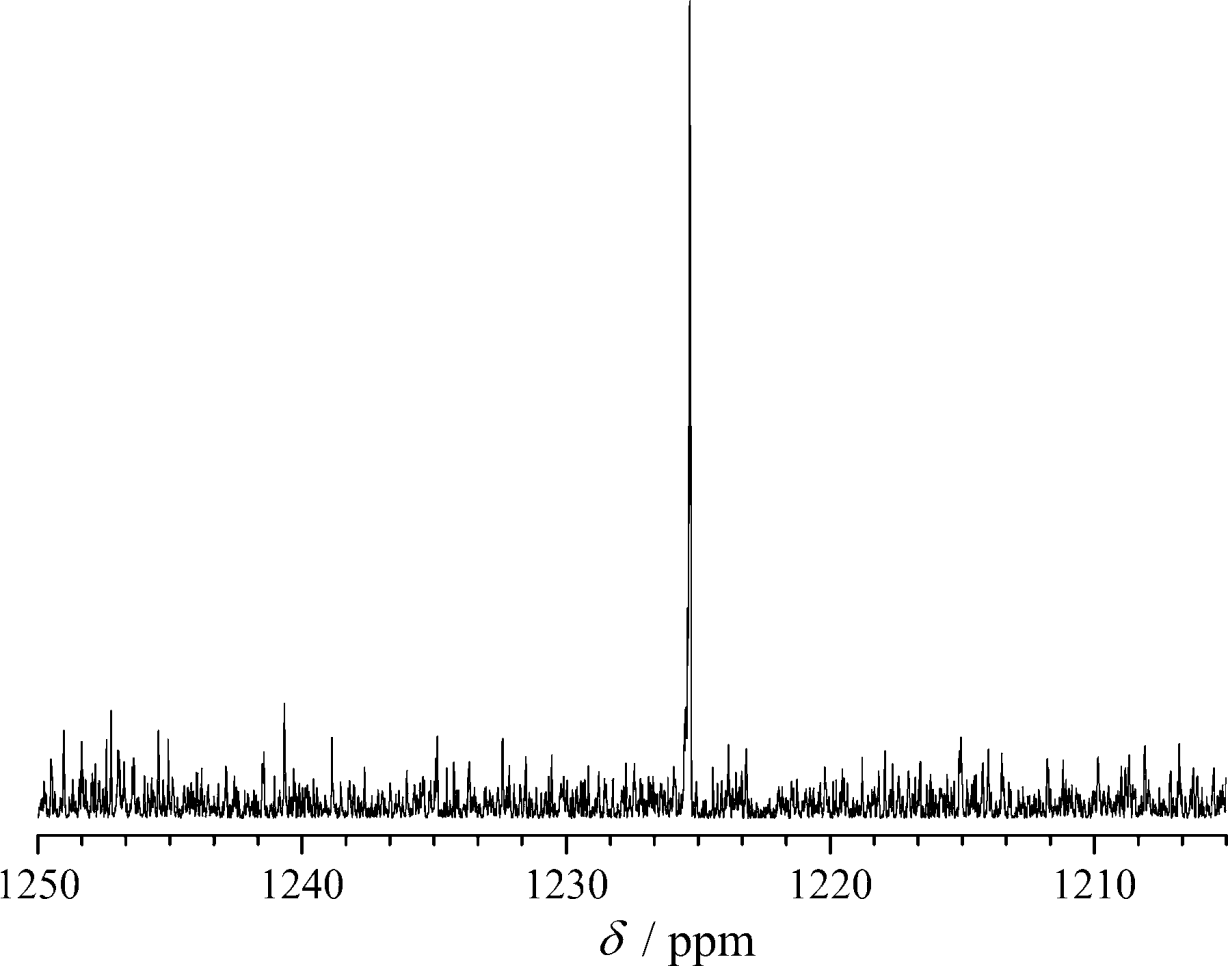
Room‐temperature ^77^Se NMR spectrum of **CsNa‐1** dissolved in 0.25 m LiOAc solution in H_2_O/D_2_O (pH 6.2).

The ^77^Se MAS NMR of **CsNa‐2** (Supporting Information, Figure S13) shows two broad signals centered at 1255 and 1187 ppm (verified for two different spinning frequencies), in line with the symmetry of **2**. Based on literature data for {Se_6_W_39_}8a and the data obtained for **CsNa‐1** (see above), we tentatively assign the upfield signal to Se^IV^ ions of the {Pd_2_SeW_7_} half of the {γ‐Pd_2_Se_2_W_13_} subunit (Figure 2 a), and the 1255 ppm peak to the Se^IV^ ions positioned in the Pd^II^‐free {SeW_6_} part of this motif. In contrast to **1**, solution ^77^Se NMR of **2** exhibits two main signals at 1316.5 ppm and 1226.8 ppm with 1.8:1 relative intensities (Supporting Information, Figure S14). The chemical shifts of the signals are evident of decomposition of the polyanions with the release of selenite ions (signal at 1316.5 ppm) concurrent with formation of **1** (singlet at 1226.8 ppm), in line with the formation of **CsNa‐1** crystals after recrystallization of **CsNa‐2** from aqueous acetate solutions. These solution stability observations for **1** and **2** are further supported by SEM images obtained after drop‐casting of 10^−4^ m **CsNa‐1** and **CsNa‐2** solutions in ultra‐pure water onto HOPG surface (Supporting Information, Figure S5).

The exact composition of ion pairs based on **1** and **2** that potentially exist in solutions and gas phase was probed by mass spectrometry. The negative‐ion‐mode ESI‐MS spectrum of **1** (Figure 4) shows a set of peaks (III–VII), which can be attributed to various ion pairs {H_*x*_Na_*y*_[Se_2_Pd_4_W_14_O_56_H]}^3−^ based on the intact polyanion **1** (Table 1), by virtue of their *m*/*z* values and analysis of the corresponding calculated and observed isotope envelopes (see Figure 4, inset; Supporting Information, Figures S17–S24). Peak II could be attributed to an ion pair based on a monovacant derivative of **1**, where one of the Pd^II^ centers is missing, while peak I belongs to a dilacunary species lacking two Pd^II^ ions with the μ_2_‐briding oxygen ion linking these metal ions together. This suggest that decomposition of **1** in gas phase (and possibly also in solution) proceeds via release of Pd^II^ centers in a first step.

**Figure 4:**
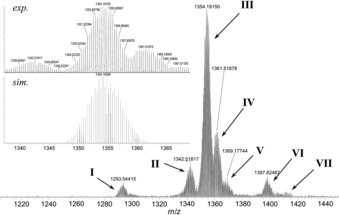
ESI mass spectrum of **1** in H_2_O/acetone (80:20 vol %) solution in negative‐ion mode. Inset: comparison of the calculated and experimentally observed isotope envelopes for the most intense signal (III).

**Table 1:** Assignment of the peaks observed in the ESI‐MS spectrum of **1**.^[a]^

Peak	Formula	*m*/*z* (calcd)	*m*/*z* (found)
I	{H_9_Na_2_[Se_2_Pd_2_W_14_O_55_]}^3−^	1293.23	1293.54
II	{H_8_Na_3_[Se_2_Pd_3_W_14_O_56_]}^3−^	1341.36	1342.52
III	{H_8_[Se_2_Pd_4_W_14_O_56_H]}^3−^	1354.18	1354.19
IV	{H_7_Na[Se_2_Pd_4_W_14_O_56_H]}^3−^	1361.51	1361.52
V	{H_6_Na_2_[Se_2_Pd_4_W_14_O_56_H]}^3−^	1368.83	1369.18
VI	{H_2_Na_6_[Se_2_Pd_4_W_14_O_56_H]}^3−^	1397.14	1397.83
VII	{Na_8_[Se_2_Pd_4_W_14_O_56_H]}^3−^ or {CsH_5_Na_2_[Se_2_Pd_4_W_14_O_56_H]}^3−^	1412.80 1412.80	1413.99

[a] Values are given for the most abundant isotopologue (see Figure 4). The small discrepancy in the experimental and calculated *m*/*z* values is due to the average element isotope composition was taken for the calculation of the masses. The precise assignment of the signals is made by comparison of the observed and calculated isotope envelopes (see the Supporting Information for details).

This is consistent with our observations of loss of Pd^II^ ions and the following POT moiety decomposition during our attempts to prepare lacunary derivatives of **1**, but also suggests that such species could in principle exist if adequately stabilized. The ESI‐MS spectrum of **2** recorded at similar conditions (Supporting Information, Figure S25) only exhibits peaks attributed to singly charged POM decomposition products (see the Supporting Information for details), consistent with our NMR observations.

In summary, we have isolated and characterized two polyanions [Se^I V^_2_Pd^II^_4_W^VI^_14_O_56_H]^11−^ and [Se^I V^_4_Pd^II^_4_W^VI^_28_O_108_H_12_]^12−^ comprising both W^VI^ and Pd^II^ addenda sites. As such, the new hybrid species bridge the conventional POT‐Pd^II^ coordination complexes and POPds. The analysis of the structural data for **CsNa‐1** suggests reactivity of μ_2_‐O ions bridging Pd^II^ ions in its {Pd_4_O_4_} fragment towards oxophilic metals. Hence, the {Pd_4_O_4_} site in **1** could serve an analogy to a vacant site of lacunary POTs, that, in combination with solution stability of **1**, could lead to a novel rich class of heterometal derivatives of mixed palladate–tungstates. On the other hand, the ESI‐MS results display a possibility for existence of lacunary species for **1** at appropriate conditions, with one or two Pd^II^ centers missing. This hypothesis is further supported by isolation of polyanion **2** which could be imagined as a dimer of two lacunary derivatives of hypothetical {γ‐Pd_4_Se_2_W_14_} species. Follow‐up work will focus on these possibilities.

## Experimental Section

**Synthesis of CsNa‐1**: Samples of Na_24_[H_6_Se_6_W_39_O_144_]⋅74 H_2_O8a (0.500 g, 0.042 mmol) and Pd(NO_3_)_2_⋅H_2_O (0.105 g, 0.423 mmol) were dissolved in 5 mL of aqueous 0.5 m NaOAc solution (prepared by addition of solid NaOH into 0.5 m HOAc solution in water until pH reaches 6.7) under vigorous stirring and heating at about 50–60 °C. The obtained clear dark‐red reaction mixture was stirred at 50 °C for 30 min and then cooled to room temperature. After that 0.5 mL of 1 m CsNO_3_ solution in H_2_O was added to the reaction mixture under stirring leading to immediate formation of light‐brown precipitate. The precipitate was collected by filtration and recrystallized from warm 0.25 m NaOAc (pH 6.7) resulting in an orange solution. Needle‐like brown‐yellow crystals of **CsNa‐1** form within several days. The filtrate produced additional portion of **CsNa‐1**, although often contaminated by hydrated Cs/Na salt of paratungstate‐*B* (based on IR and single‐crystal XRD). In this case purification is achieved by recrystallization of the obtained solid material from 0.25 m NaOAc medium (pH 6.7). The crystals of the product were collected by filtration and washed with small amount of ice cold water. Total yield: 0.177 g (33 % based on Pd).

Elemental analysis calcd (%) for C_1_H_42.5_Cs_4.3_Na_3.2_O_75_Pd_4_Se_2_W_14_: Cs 11.30, Na 1.45, Pd 8.42, Se 3.12, W 50.89; found: Cs 11.53, Na 1.51, Pd 7.89, Se 3.11, W 51.64. IR (KBr pellet), $\tilde{\nu }$ [cm^−1^]: 3424 (s, br); 1625 (m); 1420 (w); 1108 (w); 943 (s); 902 (s, sh); 874 (s); 840 (s); 819 (s); 774 (s); 713 (s), 676 (s); 502 (s); 451 (s). Raman (solid sample, *λ*_e_=1064 nm), $\tilde{\nu }$ [cm^−1^]: 958 (s); 891 (m); 872 (m); 835 (m); 787 (w); 582 (w); 507 (w, br); 241 (w, br); 197 (m); 161 (m, br); 130 (m); 100 (m); 75 (m). ^77^Se NMR (H_2_O/D_2_O): 1225.3 ppm. ^77^Se MAS NMR: 1202 ppm. UV/Vis (0.25 m NaOAc buffer solution, pH 6.7): *λ*_max_ (*ϵ*)=227 (74450), 273 (shoulder, 34153), 414 nm (1484 mol^−1^ dm^−3^ cm^−1^). CSD no.: 431484.

**Synthesis of CsNa‐2**: Na_24_[H_6_Se_6_W_39_O_144_]⋅74 H_2_O8a (0.200 g, 0.017 mmol) and Pd(NO_3_)_2_⋅H_2_O (0.026 g, 0.105 mmol) were dissolved in 2 mL of H_2_O under vigorous stirring and heating at about 50–60 °C. After the dissolution of all the reagents, the reaction mixture was stirred and further heated for 1 h and then cooled to room temperature and filtered. Three drops of 1 m aqueous CsNO_3_ solution were added to the obtained dark red–brown filtrate. The obtained pale brown precipitate^13^ was filtered and the evaporation of the resulting solution at room temperature led to brown crystalline material of **CsNa‐2** within 1–3 days. Crystals were collected by filtration, washed with ice‐cold water and dried in air. Yield: 0.040 g (17 % based on W).

Elemental analysis calcd (%) for H_72_Cs_9.5_Na_2.5_O_138_Pd_4_Se_4_W_28_: Cs 13.31, Na 0.61, Pd 4.49, Se 3.33, W 54.24; found: Cs 13.22, Na 0.61, Pd 4.49, Se 3.39, W 54.2. IR (KBr pellet), $\tilde{\nu }$ [cm^−1^]: 3423 (s, br); 1614 (s); 954 (s); 843 (s); 768 (s); 704 (s); 662 (s, br); 491 (m); 427 (s). Raman (solid sample, *λ*_e_=1064 nm), $\tilde{\nu }$ [cm^−1^]: 970 (s); 914 (m); 902 (m); 885 (m); 866 (w, sh); 812 (m); 717 (w); 660 (m); 646 (m); 513 (w); 503 (w); 216 (m); 110 (m); 75 (m). ^77^Se MAS NMR: 1255 and 1187 ppm. CSD no.: 431485.

The Supporting Information for this article includes experimental and crystallographic details, powder X‐ray diffraction, XPS/SEM data, bond valence sum values; IR, Raman, UV/Vis, ^77^Se MAS and solution NMR spectra, and ESI‐MS with simulations.

## Supporting information

As a service to our authors and readers, this journal provides supporting information supplied by the authors. Such materials are peer reviewed and may be re‐organized for online delivery, but are not copy‐edited or typeset. Technical support issues arising from supporting information (other than missing files) should be addressed to the authors.

Supplementary
