# Supplementary material for: Classical/Non‐classical Polyoxometalate Hybrids
Source: Chemistry. 2016 Oct 5;22(45):16052–6. doi: 10.1002/chem.201604238 (PMC5095796; doi:10.1002/chem.201604238)
Supplement: Supplementary file 1 — Supplementary [file CHEM-22-16052-s001.pdf]

CHEMISTRY
A European Journal

Supporting Information

Classical/Non-classical Polyoxometalate Hybrids

Natalya V. Izarova,*[a] Beatrix Santiago-Schübel,[b] Sabine Willbold,[b] Volkmar Heß,[a, c] and Paul Kögerler*[a, c]

chem_201604238_sm_miscellaneous_information.pdf

Content

I. General methods and materials…………………………………………………….… p. 2

II. X-ray crystallography……………………………………………………………….. p. 4

III. Bond valence sum calculations ………………………………………………. p. 10

IV. XPS and SEM investigations………………………………………………………. p. 13

V. Thermogravimetrical analysis…………………………………………………. p. 16

VI. Vibrational spectra…………………………………………………………………… p. 18

VII. NMR measurements……………………………………………………………. p. 19

VIII. UV-vis spectroscopy measurements……………………………………………… p. 21

IX. Electrospray mass spectrometry measurements…………………………….……… p. 23

X. References…………………………………………………………………………… p. 32

I. GENERAL METHODS AND MATERIALS

Reagents were used as purchased without further purification. $Na_{24}[H_6Se_6W_{39}O_{144}]\cdot 74H_2O$ was prepared according to the reported procedure.[1] Elemental analysis results (ICP-OES) were obtained from Central Institute for Engineering, Electronics and Analytics (ZEA-3), Forschungszentrum Jülich GmbH (D-52425 Jülich, Germany). TGA/DTA measurements were carried out with a Mettler Toledo TGA/SDTA 851 in dry N_2 (60 ml min^{-1}) at a heating rate of 5 K min^{-1}. Vibrational spectra were recorded on a Bruker VERTEX 70 FT-IR spectrometer coupled with a RAM II FT-Raman module (1064 nm Nd:YAG laser) on KBr disks for the FT-IR and the solid material for the Raman measurements. UV-Vis spectra were measured using 10 mm quartz cuvettes on an Analytik Jena Specord S600 spectrophotometer. Solution ^{77}Se NMR spectra were recorded at room temperature in 5 mm tubes using a Bruker Avance 600-MHz spectrometer equipped with a prodigy probe with resonance frequency of 76.41 MHz for ^{77}Se. Chemical shifts are reported with respect to aqueous H_2SeO_3 solution (^{77}Se, $\delta = 1302$ ppm) as an external reference. Solid-state ^{77}Se MAS NMR experiments were performed at ambient temperature on a Varian NMR System NB 600 equipped with a 4.0 mm HXY Triple Resonance MAS probe head and operating at 114.51 MHz for ^{77}Se. The magic angle spinning (MAS) ^{77}Se-NMR spectra were recorded at a spinning frequency of 15 kHz and a recycle delay of 60 s. Solid Na_2SeO_3 was used as the external reference ($\delta = 1274$ ppm). The observed isotropic peaks were further verified by changing the spinning frequency to 17 kHz for **CsNa-2**.

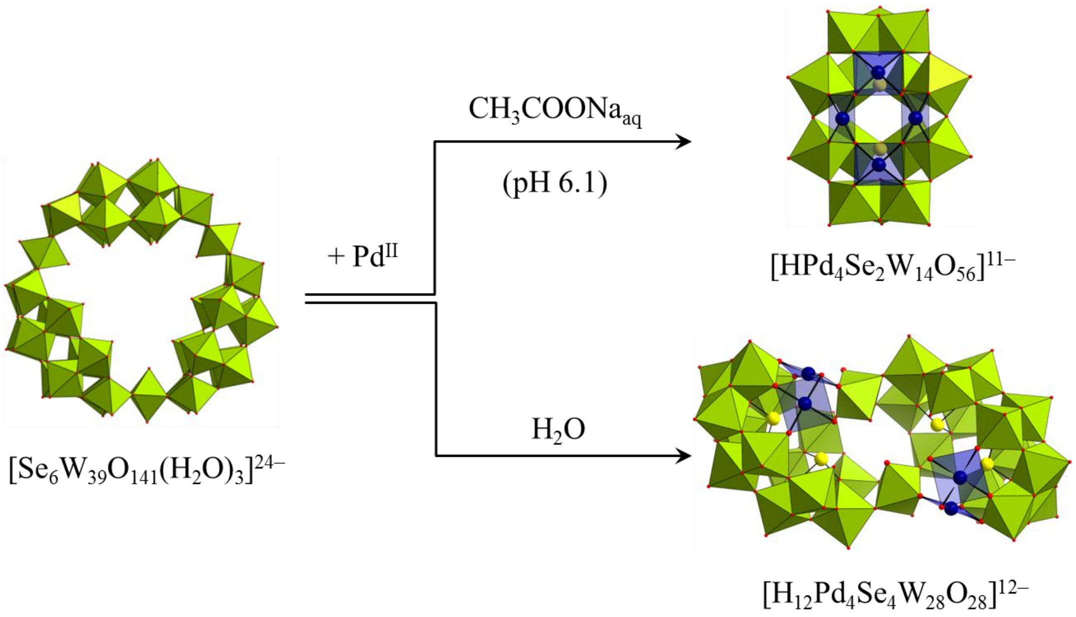

Scheme S1. Schematic representation of synthetic pathways for preparation **1** and **2**. Color legend: WO_6, lime green octahedra; PdO_4, transparent-blue squares; Pd, dark-blue, Se, yellow and O, red spheres.

II. X-RAY CRYSTALLOGRAPHY

II.1. Single-crystal X-ray diffraction data for **CsNa-1** and **CsNa-2** were collected on a SuperNova (Agilent Technologies) diffractometer with MoKα radiation (λ = 0.71073 Å) at 120 K. The crystals were mounted in a Hampton cryoloop with Paratone-N oil to prevent water loss. Absorption corrections were applied numerically based on multifaceted crystal model using CrysAlis software.[2] The SHELXTL software package[3] was used to solve and refine the structure. The structures were solved by direct methods and refined by full-matrix least-squares method against $|F|^2$ with anisotropic thermal parameters for all heavy atoms (Cs, Na, Pd, Se and W). No hydrogen atoms of crystal water molecules or OH groups of the polyanions have been located. The relative site occupancy factors for the disordered positions of caesium and sodium cations as well as oxygen atoms of co-crystallized water molecules were first refined in an isotropic approximation with U_{iso}= 0.05 and then fixed at the obtained values and refined without thermal parameter restrictions. Due to severe disorder we could only locate 2.5 Na^+ cations in the structure of **CsNa-1** while 3 Na^+ counterions are present in the structure based on elemental analysis and charge balance considerations. Similarly, we could only locate 1.625 out of 2.5 Na^+ as well as 9.05 out of 9.5 Cs^+ countercations in the structure of **CsNa-2** (with occupancy as low as 5 % for some of the Cs^+ positions). We also found less crystal water molecules in single-crystal X-ray analysis compared to the number of co-crystallized H_2O determined from elemental and thermogravimetrical analyses (12 *vs*. 18 for **CsNa-1** and 19 *vs*. 30 for **CSNa-2**, respectively), which also reflects the high degree of solvent disorder in the solid-state lattices of **CsNa-1** and **CsNa-2**. This is consistent with large solvent-accessible volume remaining in the structures. For the overall consistency, the final formulae in the CIFs correspond to the composition of the bulk materials determined by elemental analysis and TGA and in the case of **CsNa-1** also include small amounts of Cs and Na acetate impurities not identifiable in single-crystal X-ray diffraction, as all further studies were performed on the isolated well-dried bulk materials of **CsNa-1** and **CsNa-2**.

The rather high value of R_{int} for **CsNa-1** (0.135) combined with several violations of systematic absences are consistent with the twinning issue (especially considering the needle-like shape of the crystals). As we were unable to discern any pattern in the list of reflections for which F(obs) ≫ F(calc), we were not able to systematically remove composite reflections. The twinning issue is most likely also the reason for the appearance of a rather unusual electron density maximum (7.86 e Å^{-3}) in the structure of **CsNa-2** for which no any reasonable assignment could be suggested due to very small distances between the electron density maximum to O6D (1.5 Å) and O36T (1.7 Å). It also cannot be a ligand on any metal

center as the closest distance from this electron density peak is 3.07 Å to Pd6 or 3.15 Å to W36. We have tried to measure several other crystals of **CsNa-2**, however, while this unusual electron density maximum was absent in some other data, these datasets presented other serious problems due to the twinning issue, resulting in higher *R* factors. The attempts to split different twinning components also did not result in a data set of a sufficient quality.

Additional crystallographic data are summarized in Table S1. The main bond lengths in **1** and **2** are shown in Table S2. Further details on the crystal structure investigation may be obtained from Fachinformationszentrum Karlsruhe, 76344 Eggenstein-Leopoldshafen, Germany [fax (+49) 7247-808-666; e-mail crysdata@fiz-karlsruhe.de), upon quoting the depository numbers CSD 431484 (**CsNa-1**) and CSD 431485 (**CsNa-2**).

II.2. Powder X-ray diffraction (PXRD) data (Fig. S1 and S2) were recorded on a Stoe Stadi P diffractometer (Stoe&Cie, Darmstadt) in transmission mode (CuKα_1 radiation, Ge monochromator) with an image plate detector (140° in 2θ, stepwidth 0.015°). Scanning time was 2 h for the whole pattern.

Table S1. Crystal data and structure refinement for **CsNa-1** and **CsNa-2**

Sample	**CsNa-1**	**CsNa-2**
Empirical formula	$C_1Cs_{4.3}H_{42.5}Na_{3.2}O_{75}Pd_4Se_2W_{14}$	$Cs_{9.5}H_{72}Na_{2.5}O_{138}Pd_4Se_4W_{28}$
Formula weight, g/mol	5057.35	9489.94
Crystal system	Orthorhombic	Triclinic
Space group	*Pnnm*	*P*–1
a / Å	17.1654(5)	20.5433(6)
b / Å	19.4913(8)	22.6350(5)
c / Å	21.2392(5)	35.6622(10)
α	90°	79.842(2)°
β	90°	87.995(2)°
γ	90°	67.256(3)°
Volume / Å^3	7106.1(4)	15043.5(7)
Z	4	4
D_{calc}, g/cm^3	4.727	4.190
Absorption coefficient, mm^{-1}	26.879	25.122
F(000)	8833	16472
Crystal size, mm	0.04 × 0.15 × 0.22	0.07 × 0.10 × 0.13
Theta range for data collection	4.13° – 25.02°	4.08° – 24.11°
Completeness to Θ_{max}	99.3 %	95.9 %
Index ranges	$-20 < h < 20$,<br>$-22 < k < 23$,<br>$-25 < l < 21$	$-23 < h < 23$,<br>$-26 < k < 26$,<br>$-40 < l < 40$
Reflections collected	33984	133640
Independent reflections	6428	45916
R_{int}	0.1352	0.0694
Observed ($I > 2\sigma(I)$)	3627	33377
Absorption correction	Empirical using spherical harmonics	
T_{min} / T_{max}	0.0794 / 0.4250	0.0649 / 0.2738
Data / restraints / parameters	6428 / 6 / 288	45916 / 49 / 2104
Goodness-of-fit on F^2	1.062	1.045
R_1, wR_2 ($I > 2\sigma(I)$)	$R_1 = 0.0623$,<br>$wR_2 = 0.1179$	$R_1 = 0.0500$,<br>$wR_2 = 0.1113$
R_1, wR_2 (all data)	$R_1 = 0.1239$,<br>$wR_2 = 0.1519$	$R_1 = 0.0795$,<br>$wR_2 = 0.1285$
Largest diff. peak and hole / e Å^{-3}	3.103 / –2.676	7.868 / –2.899

Table S2. Bond lengths in **1** and **2** as determined from **CsNa-1** and **CsNa-2**.

**Bond type**	**Bond length / Å**	
	**CsNa-1**	**CsNa-2**
Se–μ_4-O	1.717(14)	1.729(15) – 1.741(15)
Se–μ_3-O	1.677(16) – 1.725(15)	1.665(13) – 1.724(13)
Pd–O_{term}	–	2.007(18) – 2.040(18)
Pd–μ_2-O (Pd–O–Pd)	1.976(14) – 2.010(15)	2.011(14) – 2.039(13)
Pd–μ_2-O (Pd–O–W)	1.986(17) – 2.009(15)	1.949(16) – 2.018(15)
W=O_{term}	1.686(15) – 1.751(16)	1.684(14) – 1.790(14)
W–O_{term}	–	2.236(14) – 2.311(16)
W–μ_2-O (W–O–Pd)	1.770(18) – 1.814(17)	1.764(14) – 2.063(14)
W–μ_2-O (W–O–W)	1.855(16) – 2.044(18)	1.803(14) – 2.116(15)
W–μ_3-O (Se, 2 W)	2.280(16) – 2.340(17)	2.257(13) – 2.437(12)
W–μ_4-O (Se, 3W)	2.380(16) – 2.408(16)	2.383(13) – 2.452(13)

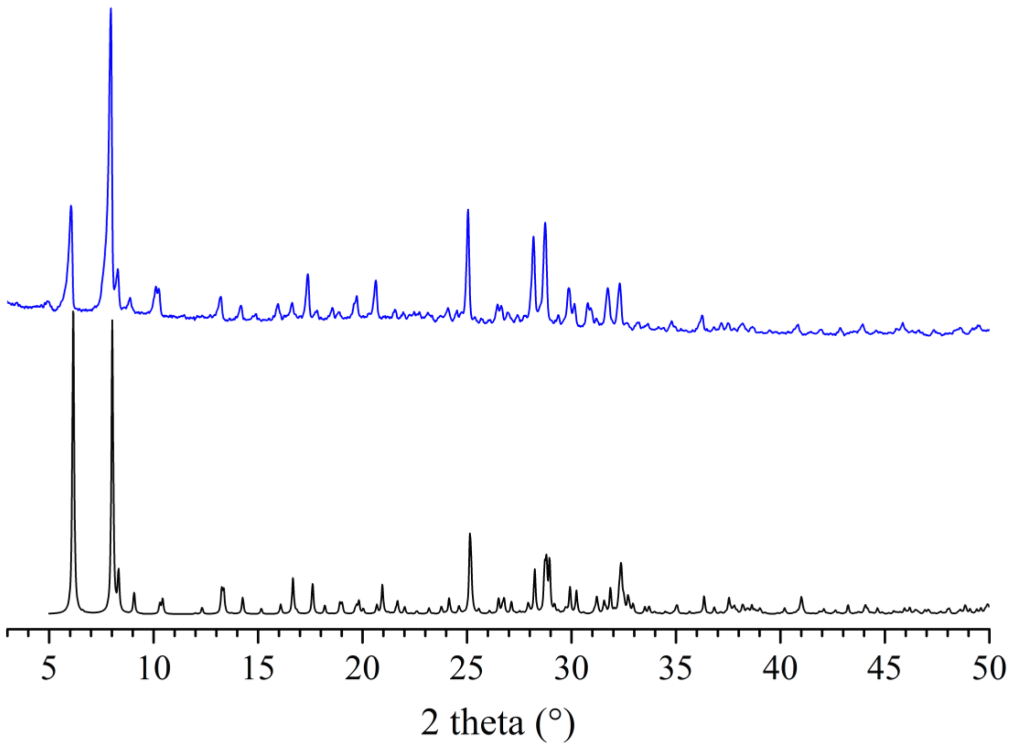

Figure S1. Experimental (blue) and calculated from the single-crystal structure data (black) powder X-ray diffraction patterns for **CsNa-1**.

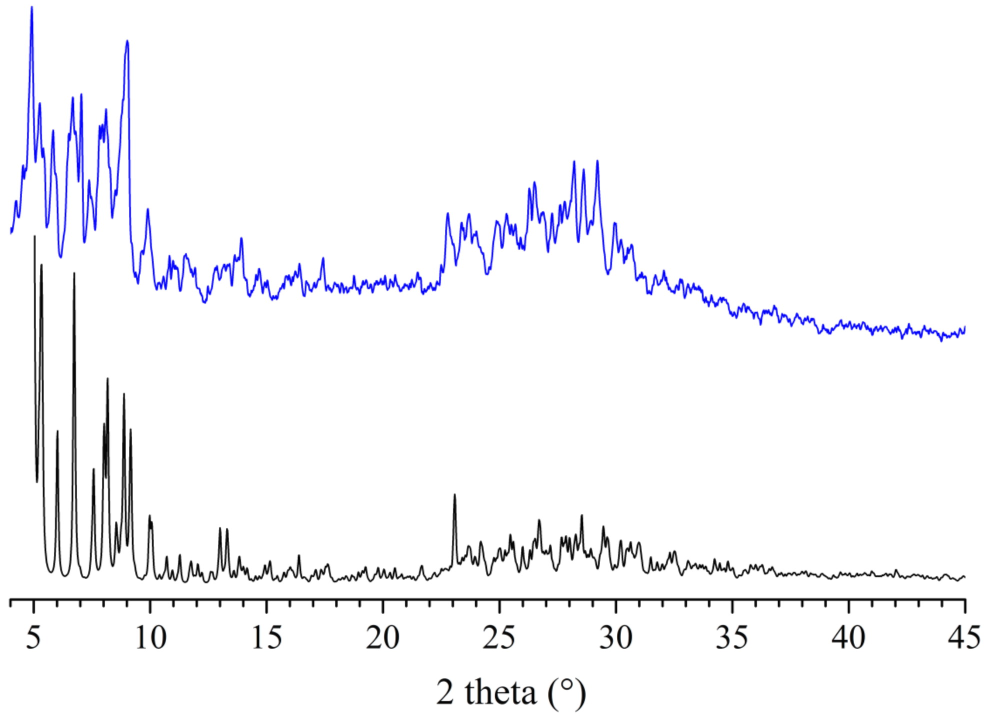

Figure S2. Experimental (blue) and calculated from the single-crystal structure data (black) powder X-ray diffraction patterns for **CsNa-2**.

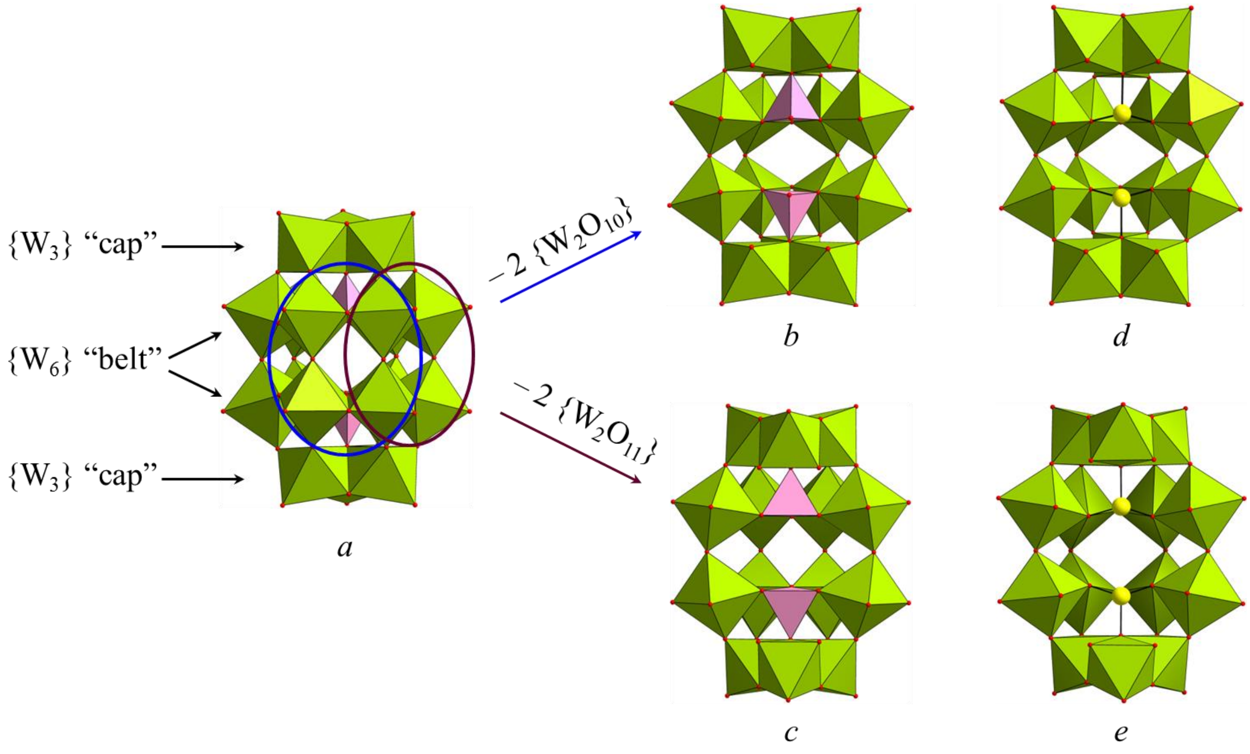

Figure S3. Formal decomposition of the parental {α-P_2W_{18}} structure (*a*) leading to a hypothetical (*b*) and previously observed[4] (*c*) {α-P_2W_{14}} fragments. Comparison with {α-Se_2W_{14}} (*d*) and {γ-Se_2W_{14}} (*e*) units. Color code: WO_6, lime green octahedra, PO_4, pink tetrahedra, Se yellow and O red spheres. The blue and dark-red circles highlight the pairs of {W_2O_{10}} and {W_2O_{11}} groups, absent in the {α-P_2W_{14}} moieties.

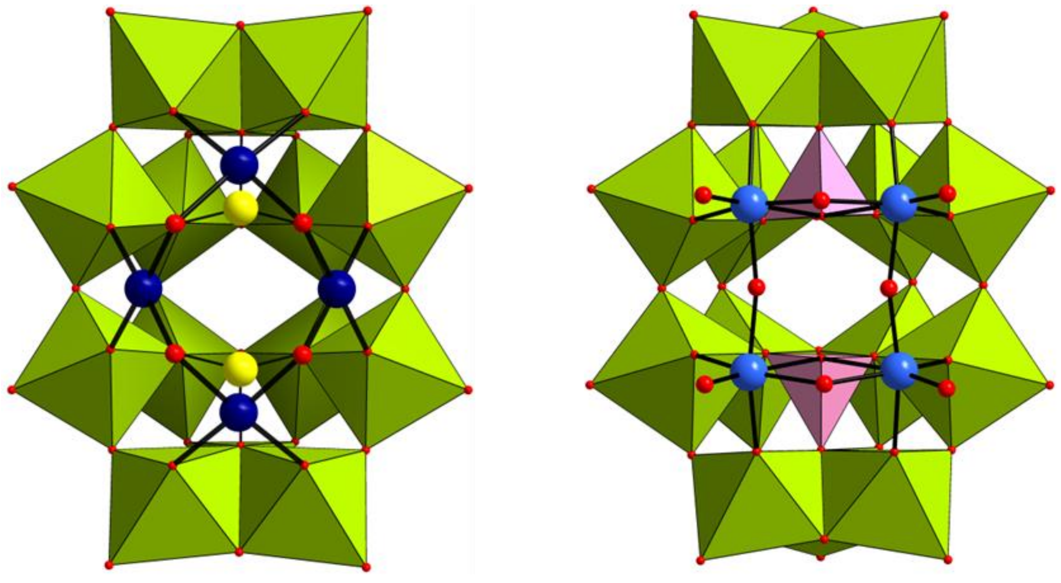

Figure S4. Comparison of {α-$Se_2Pd_4W_{14}$} (**1**, left) and {α-P_2W_{18}} [5] (right) structures. Color legend: WO_6, lime green octahedra; Pd, blue, W, light blue, Se, yellow, O, red spheres.

III. BOND VALENCE SUM CALCULATIONS

Bond valence sum calculations[6] for **CsNa-1** (Table S3) and **CsNa-2** (Table S4) are consistent with the +VI oxidation state for all W, +IV for all Se and +II for all Pd centers in **1** and **2**. The calculated BVS values for oxygen atoms in polyanions **1** indicate a possibility of a presence of one proton associated with the polyanion, which is disordered over four μ_2-O atoms of {Pd_4O_4} moiety (O13D and O23D). The BVS values for oxygen atoms in **2** confirm the presence of terminal aqua ligands on Pd^{II} centers (O1D, O4D, O6D, O8D) as well as within the *trans*-{O=W(H_2O)} groups (O14H, O15H, O42H and O43H). Moreover, the oxo ligands *trans* to the terminal aqua ligands on Pd^{II} centers (O1D1, O8D4, O9D6, O6D8) as well as the μ_2-bridging oxygens connecting two Pd^{II} ions in the structure of 2 (O12D, O34D, O56D and O78D) seem to be monoprotonated. The number of protons found by BVS method is fully consistent with that determined from elemental analyses.

Table S3. Bond valence sum values for different atoms in **CsNa-1**

**W, Se, and Pd centers**		**μ_2-O (W–O–W)**		**Terminal oxygens**	
W1	6.06	O12	–1.87	O1T	–1.82
W2	6.44	O13	–1.84	O2T	–2.04
W3	5.92	O14	–2.12	O3T	–1.85
W4	6.31	O23	–1.91	O4T	–1.86
W5	6.36	O25	–2.11	O5T	–1.84
W6	6.01	O26	–2.13	O6T	–1.75
W7	6.23	O37	–2.12	O7T	–1.82
Se1	4.09	O44	–2.07	**μ_2-O (Pd–O–W)**	
Pd1	2.30	O45	–1.95	O1D2	–2.06
Pd2	2.28	O55	–2.08	O3D3	–2.08
Pd3	2.36	O56	–2.04	O4D1	–2.12
**Oxygens of {SeO_3} group**		O66	–2.03	O7D2	–2.06
O1S1	–2.15	O67	–1.89	**μ_2-O (Pd–O–Pd)**	
O2S1	–1.97	O77	–2.06	O13D	–1.33
O3S1	–2.11			O23D	–1.29

Table S4 (part 1). Bond valence sum values for different atoms in **CsNa-2**

**W centers**		**W centers**		**Terminal oxygens (W)**			
W1	6.09	W38	6.32	O1T	–2.02	O35T	–1.82
W2	6.20	W39	6.30	O2T	–1.72	O36T	–1.47
W3	6.32	W40	6.27	O3T	–1.97	O36H	–1.77
W4	6.14	W41	6.15	O4T	–1.98	O37T	–1.90
W5	6.28	W42	6.20	O5T	–1.85	O38T	–2.10
W6	6.35	W43	6.40	O6T	–1.92	O39T	–1.89
W7	6.17	W44	6.25	O7T	–1.92	O40T	–1.70
W8	6.20	W45	6.20	O8T	–1.67	O41T	–1.92
W9	6.20	W46	6.22	O8H	–2.16	O42T	–1.89
W10	6.19	W47	6.50	O9T	–1.69	O42H	–0.42
W11	6.38	W48	6.27	O10T	–2.01	O43T	–1.81
W12	6.24	W49	6.27	O11T	–2.21	O43H	–0.50
W13	6.10	W50	6.19	O12T	–2.00	O44T	–2.06
W14	6.37	W51	6.18	O13T	–1.96	O45T	–2.06
W15	6.21	W52	6.33	O14T	–1.73	O46T	–1.91
W15A	6.48	W53	6.24	O14H	–0.56	O47T	–1.94
W16	6.31	W54	6.11	O15T	–1.78	O48T	–1.86
W17	6.23	W55	6.29	O15H	–0.55	O49T	–1.64
W18	6.44	W56	6.17	O16T	–2.04	O49H	–1.94
W19	6.20	**Se centers**		O17T	–1.86	O50T	–1.87
W20	6.29	Se1	4.12	O18T	–1.89	O51T	–1.77
W21	6.27	Se2	4.33	O19T	–1.86	O52T	–1.87
W22	6.38	Se3	4.05	O20T	–1.97	O53T	–2.02
W23	6.27	Se4	4.19	O21T	–1.76	O54T	–1.75
W24	6.39	Se5	4.08	O21H	–1.67	O55T	–1.89
W25	6.36	Se6	4.03	O22T	–1.80	O56T	–1.95
W26	6.27	Se7	4.16	O23T	–1.83	**Terminal oxygens (Pd)**	
W27	6.11	Se8	4.07	O24T	–1.90		
W28	6.23	**Pd centers**		O25T	–1.83	O1D	–0.56
W29	6.16			O26T	–1.80	O4D	–0.59
W30	6.24	Pd1	2.27	O27T	–1.79	O6D	–0.68
W31	6.28	Pd2	2.38	O28T	–1.80	O8D	–0.54
W32	6.41	Pd3	2.38	O29T	–1.81	**μ_2-O (Pd–O–Pd)**	
W33	6.23	Pd4	2.25	O30T	–2.07		
W34	6.33	Pd5	2.40	O31T	–2.00	O12D	–1.21
W35	6.16	Pd6	2.23	O32T	–1.85	O34D	–1.08
W36	6.20	Pd7	2.40	O33T	–1.95	O56D	–1.15
W37	6.49	Pd8	2.26	O34T	–1.81	O78D	–1.23

Table S4 (part 2). Bond valence sum values for different atoms in **CsNa-2**

μ_2-O (Pd–O–W)		μ_2-O (W–O–W)		μ_2-O (W–O–W)		μ_2-O (W–O–W)	
O1D1	–1.28	O113	–2.19	O323	–1.97	O523	–2.00
O4D1	–1.96	O115	–2.12	O326	–2.18	O525	–2.03
O1D2	–2.03	O123	–2.03	O334	–2.10	O535	–2.07
O5D2	–2.10	O124	–2.19	O337	–2.14	O545	–1.95
O7D2	–2.04	O134	–2.06	O345	–1.90	O546	–2.16
O2D3	–2.12	O148	–2.17	O348	–2.18	O556	–1.88
O4D3	–2.07	O156	–2.03	O359	–2.15	O610	–2.19
O8D3	–2.07	O157	–2.08	O360	–2.07	O711	–2.13
O5D4	–2.07	O167	–2.05	O367	–1.86	O812	–2.10
O8D4	–1.37	O168	–2.16	O370	–2.02	O910	–2.12
O3D5	–2.16	O169	–2.15	O378	–2.10	O912	–1.99
O5D5	–1.98	O170	–2.14	O381	–2.14	**Oxygens of {SeO_3} group**	
O9D5	–1.99	O171	–2.09	O389	–2.15		
O2D6	–2.11	O182	–2.16	O391	–2.19	O1S1	–2.01
O9D6	–1.47	O189	–1.96	O393	–2.17	O2S1	–2.08
O0D7	–2.15	O190	–2.12	O401	–1.97	O3S1	–2.03
O2D7	–2.14	O193	–2.11	O402	–2.17	O1S2	–2.06
O6D7	–2.09	O201	–1.88	O401	–2.07	O2S2	–2.18
O3D8	–1.95	O204	–2.19	O426	–2.11	O3S2	–2.11
O6D8	–1.26	O215	–2.16	O434	–2.12	O1S3	–1.96
**μ_2-O (W–O–W)**		O223	–1.95	O435	–2.23	O2S3	–2.02
		O226	–2.07	O445	–1.99	O3S3	–2.03
O12	–1.92	O234	–2.15	O446	–2.13	O1S4	–2.04
O13	–2.09	O236	–2.02	O447	–2.03	O2S4	–2.10
O23	–1.97	O245	–1.91	O458	–2.05	O3S4	–2.04
O24	–2.06	O247	–2.03	O459	–2.16	O1S5	–2.04
O25	–2.05	O257	–2.06	O460	–2.15	O2S5	–1.99
O36	–2.16	O267	–1.97	O467	–1.92	O3S5	–2.07
O37	–2.03	O268	–1.96	O471	–2.09	O1S6	–2.05
O45	–2.02	O278	–2.06	O478	–2.10	O2S6	–2.07
O48	–2.19	O290	–1.99	O482	–2.13	O3S6	–1.99
O56	–2.15	O291	–1.96	O489	–2.03	O1S7	–2.11
O59	–2.07	O301	–1.97	O493	–2.21	O2S7	–2.08
O67	–2.03	O302	–2.08	O501	–1.97	O3S7	–1.99
O89	–2.03	O303	–2.05	O504	–2.02	O1S8	–2.06
O101	–2.10	O314	–2.05	O512	–2.15	O2S8	–2.00
O103	–2.08	O315	–2.04	O514	–2.01	O3S8	–2.01

IV. XPS AND SEM INVESTIGATIONS

1. Surface deposition.

Compounds **1** and **2** have been deposited on HOPG by drop-casting of 10^{-4} M **CsNa-1** and **CsNa-2** solutions in ultra-pure water. A drop of approximately 75 µL was deposited on a freshly cleaved HOPG surface and allowed to evaporate. Afterwards the samples were transferred into an ultra-high vacuum system and investigated by scanning electron microscopy (SEM) and X-ray photoelectron spectroscopy (XPS).

2. SEM investigation.

SEM was used to investigate the morphology of the samples after drop-casting. It shows that **CsNa-1** recrystallizes in long needles (which is a typical shape for **CsNa-1** crystals) with a length of several (1 to 10) µm and a width of several hundred nm (Fig. S5, (a)-(c)). This is in full agreement with our further observation on the possibility to recrystallize **CsNa-1** from various aqueous media. The crystals are randomly oriented on the HOPG surface.

In contrast, **CsNa-2** (Fig. S5, (d)-(f)) does not appear to recrystallize. Instead spherical particles with a diameter ranging from 100 nm to 1 µm are found. The reason for this different behavior might originate from solution instability of polyanions **2**.

3. XPS investigation.

The chemical composition of **1** and **2** deposited on HOPG was investigated by XPS. A lab-based Mg Kα radiation source (hν = 1253.6 eV) in combination with a hemispherical analyzer was used. The C 1*s* peak from the HOPG substrate was set to 284.5 eV for calibrating the measurements. All measurements were performed at room temperature.

The XPS study reveals the presence of all elemental components of **1** and **2**. The W 4*f* and Se 3*d* peaks are shown in Fig. S6. The W $4f_{7/2}$ peak is detected at a binding energy of 35 eV for both species. This value is in good agreement with measurements on bulk WO_3, where W exhibits the same oxidation state +VI as in polyanions **1** and **2**. When comparing the Se 3*d* peaks a shift to higher binding energies is detected in species **2**.

The Pd 3*d* peak is shown in Fig. S7. The measurement on polyanions **1** exhibits a high signal-to-noise ratio and reveals a single Pd $3d_{5/2}$ peak at a binding energy of 337 eV, which is typical for Pd^{II} in an oxygen environment. In contrast, the measurement on species **2** exhibits a low signal-to-noise ratio. However, a shoulder of the Pd $3d_{5/2}$ peak to lower binding energies can be clearly distinguished, indicating that Pd is present in two chemical environments. A shift to lower binding energies usually results from a metallic chemical environment and thus could support a hypothesis about partial decomposition of polyanions **2** during surface deposition.

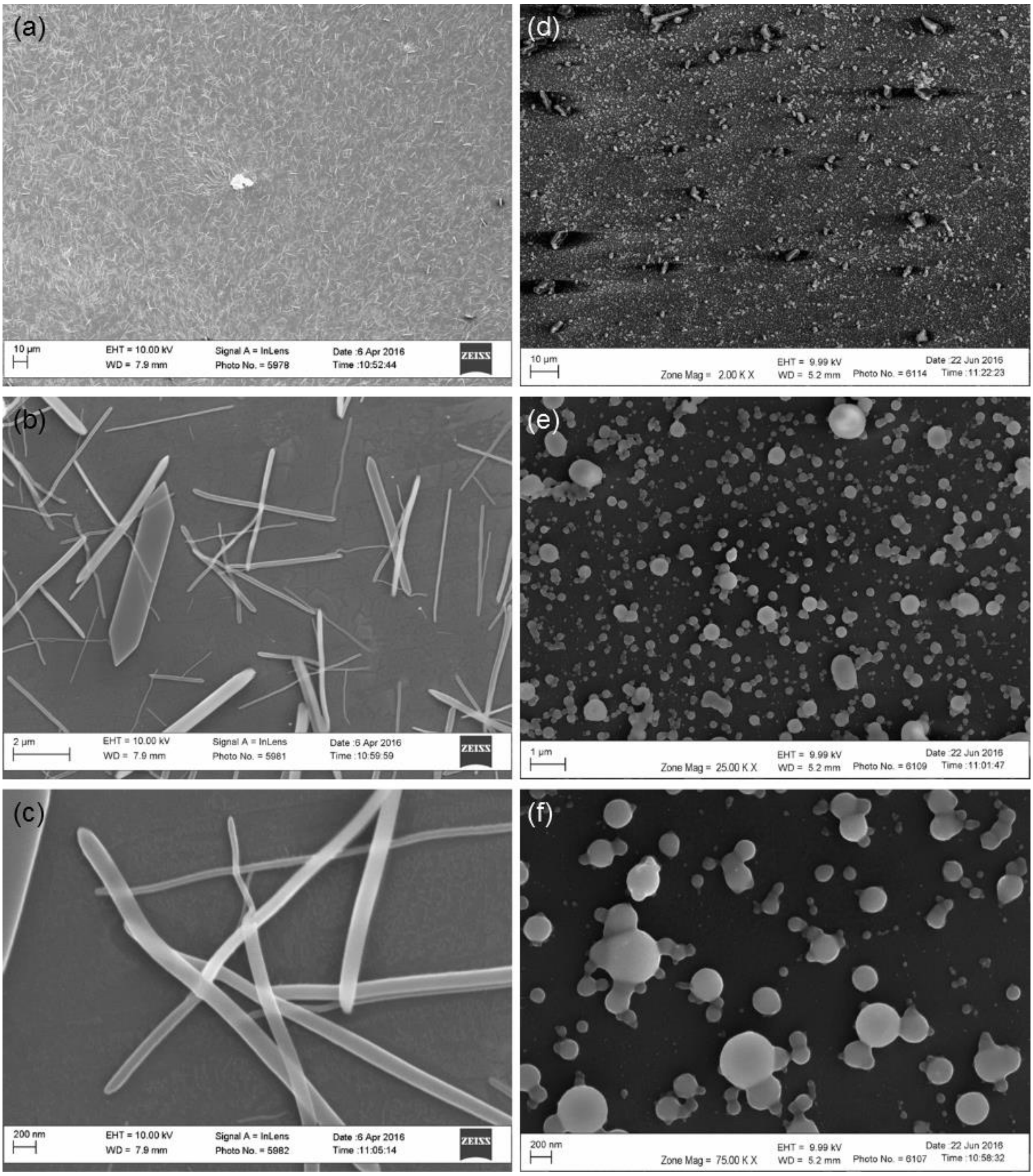

Figure S5. SEM investigation of **CsNa-1** ((a)-(c)) and **CsNa-2** solutions deposited on HOPG. Magnification in (a) 1kX, (b) 20 kX, (c) 80 kX, (d) 2 kX, (e) 25 kX, (f) 75 kX.

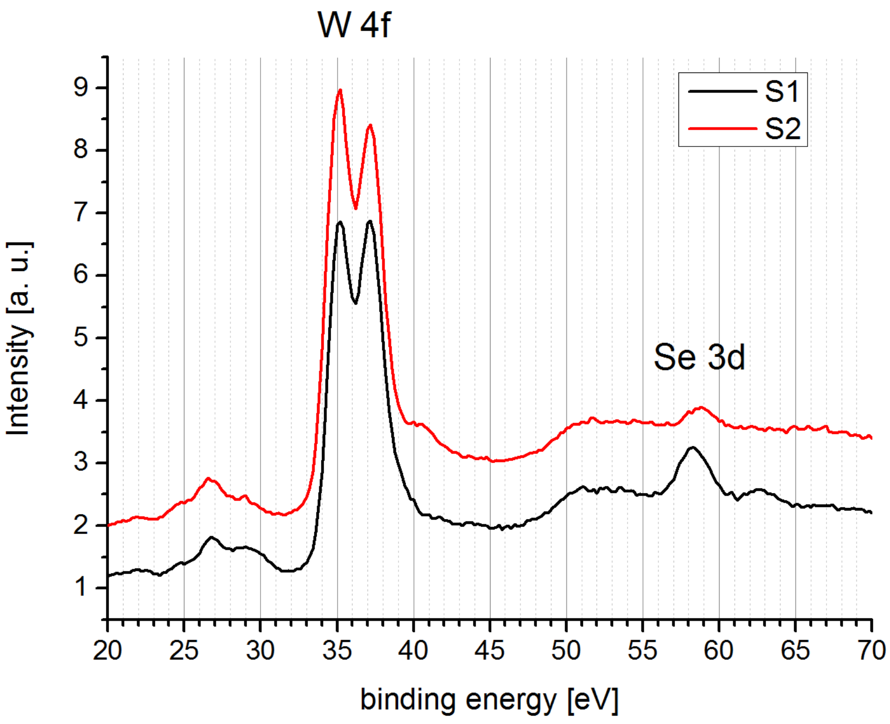

Figure S6. XPS spectra showing the W 4*f* and Se 3*d* peaks for **1** (black line) and **2** (red line).

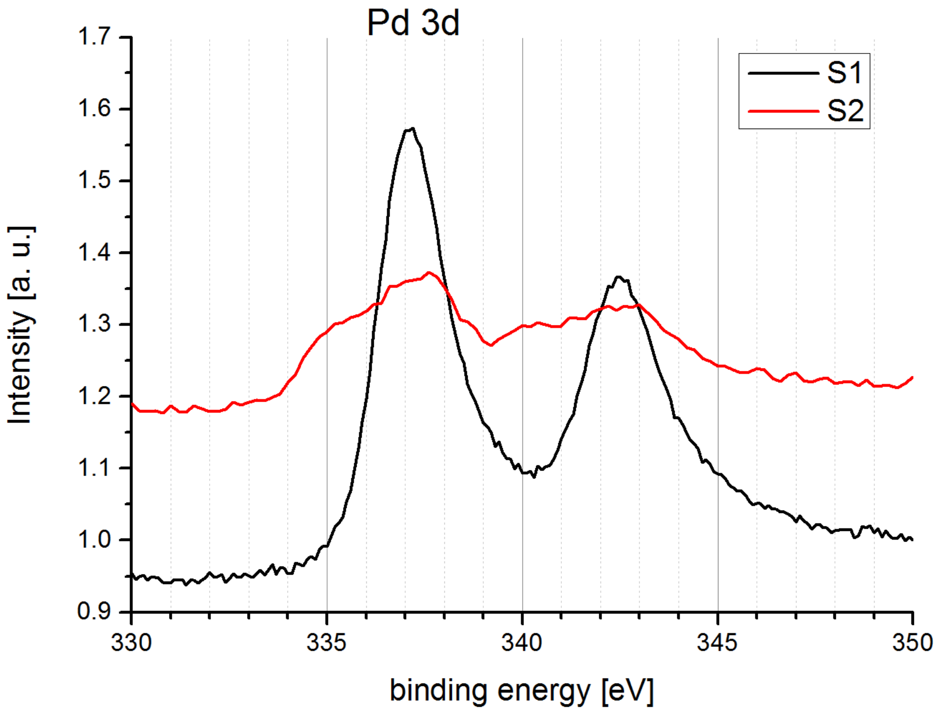

Figure S7. XPS spectra showing the Pd 3*d* peaks for **1** (black line) and **2** (red line).

V. THERMOGRAVIMETRIC ANALYSIS

1. Thermogravimetric analysis for CsNa-1 (RT to 900 °C, N_2 flux).

The TGA curve of **CsNa-1** (Fig. S8) exhibits a set of weight release steps in the temperature range 25 – 800 °C. The first sharp weight loss occurs between 25 – 100 °C and can be attributed to the removal of 15 co-crystallized water molecules per formula unit (5.3 calc. *vs*. 5.5 % observed). The broad weight loss step in the temperature range 100 – 400 °C apparently is due to the release of the remaining crystal waters as well as protons in the form of H_2O (2.0 calc. *vs*. 2.6 % observed). The following set of weight loss steps in the temperature range 400 – 800 °C should correspond to the loss of SeO_2 groups and decomposition of acetate impurity (5.0 calc. *vs*. 5.7 % observed). The total weight loss at 900 °C is 14.0 %.

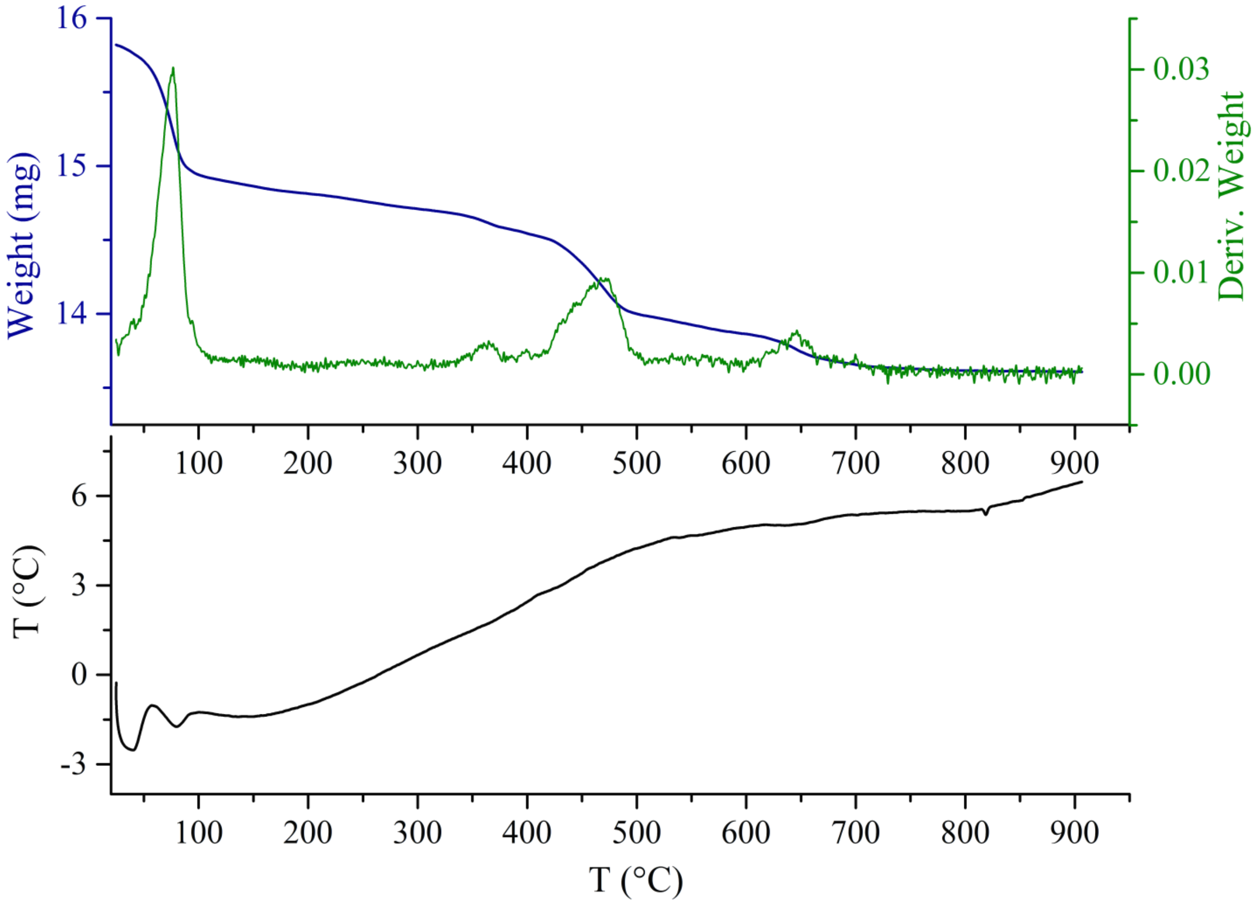

Figure S8. TGA (blue) and first derivative (green), and SDTA (black) curves for **CsNa-1** from room temperature to 900 °C under N_2 atmosphere.

2. Thermogravimetric analysis for CsNa-2 (RT to 900 °C, N_2 flux).

The TGA curve of **CsNa-2** (Fig. S9) shows several successive weight loss steps in the temperature interval 25 – 750 °C. The first set of weight loss steps occurs in the temperature interval 25 – 300 °C and corresponds to a release of crystal water molecules (5.7 calc. *vs*. 5.4 % observed). The successive weight loss between 300 and 750 °C with an exothermic peak at 432 °C in the SDTA curve corresponds to removal of 4 SeO_2 groups as well as 12 protons (either in form of H_2O or H_2SeO_3 + H_2O) per polyanion (5.8 calc. *vs*. 5.4 % observed). The total weight loss observed at 900 °C is 10.9 %.

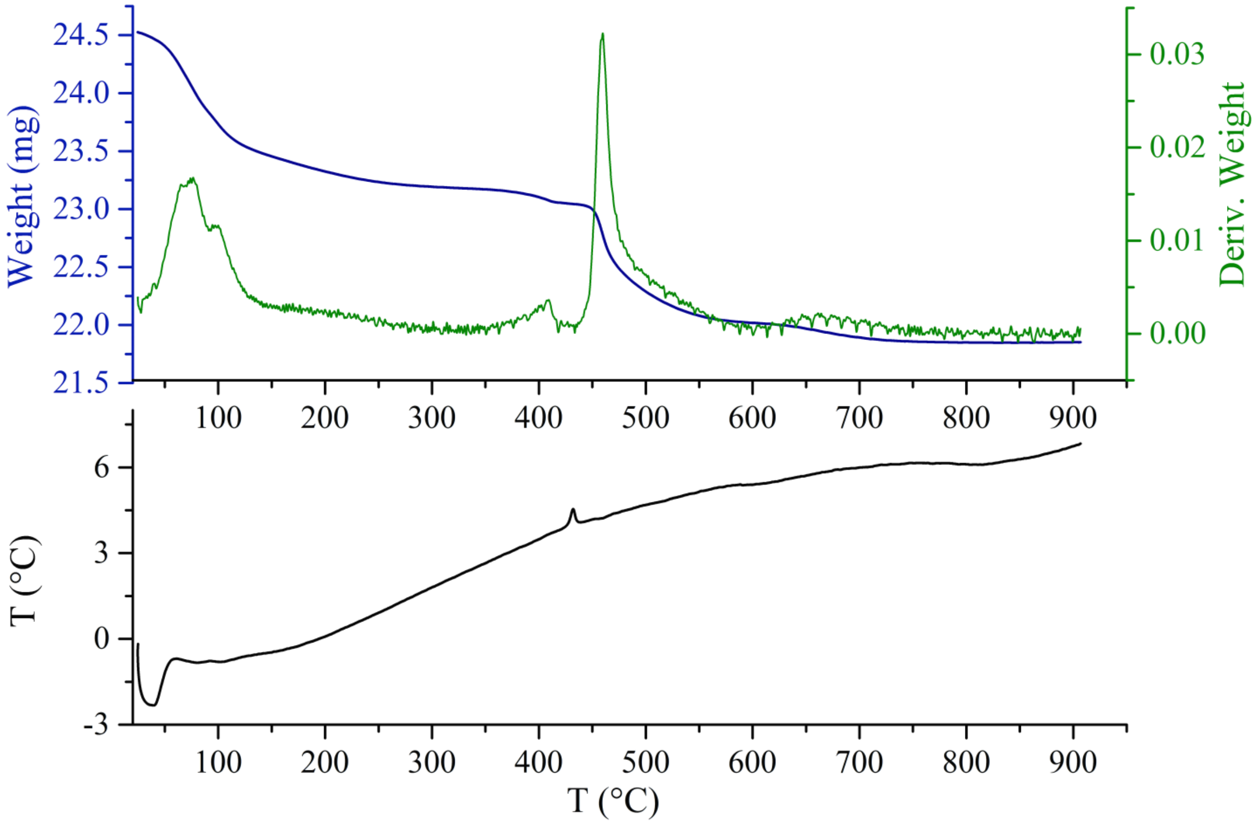

Figure S9. TGA (blue) and first derivative (green), and SDTA (black) curves for **CsNa-2** from room temperature to 900 °C under N_2 atmosphere.

VI. VIBRATIONAL SPECTRA

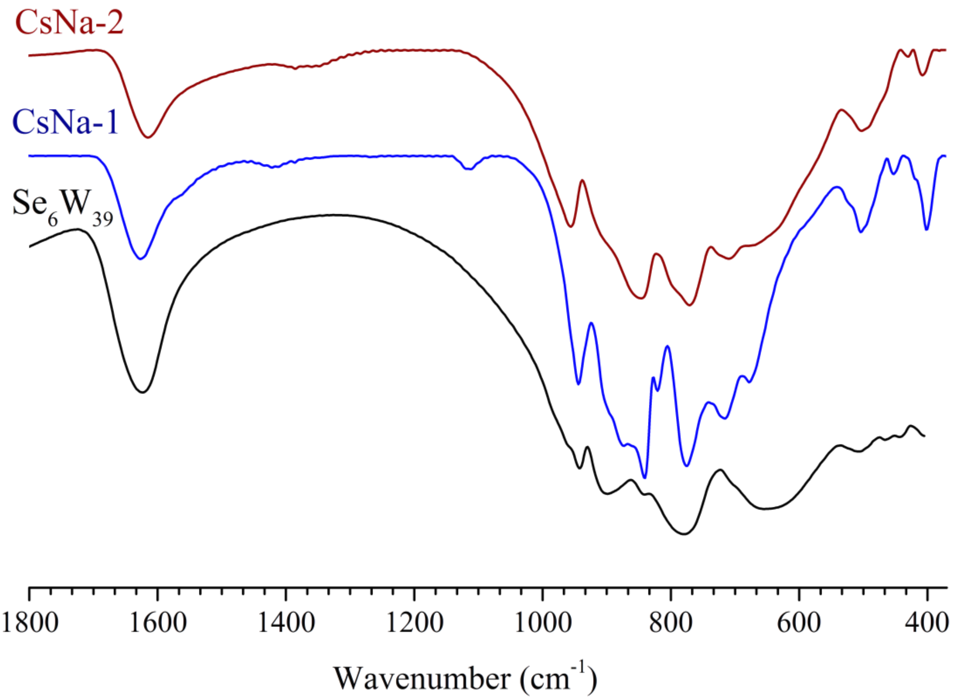

Figure S10. FT-IR spectra of **CsNa-1** (blue) and **CsNa-2** (brown) in comparison with that of the $Na_{24}[H_6Se_6W_{39}O_{144}]\cdot 74H_2O$ precursor (black).

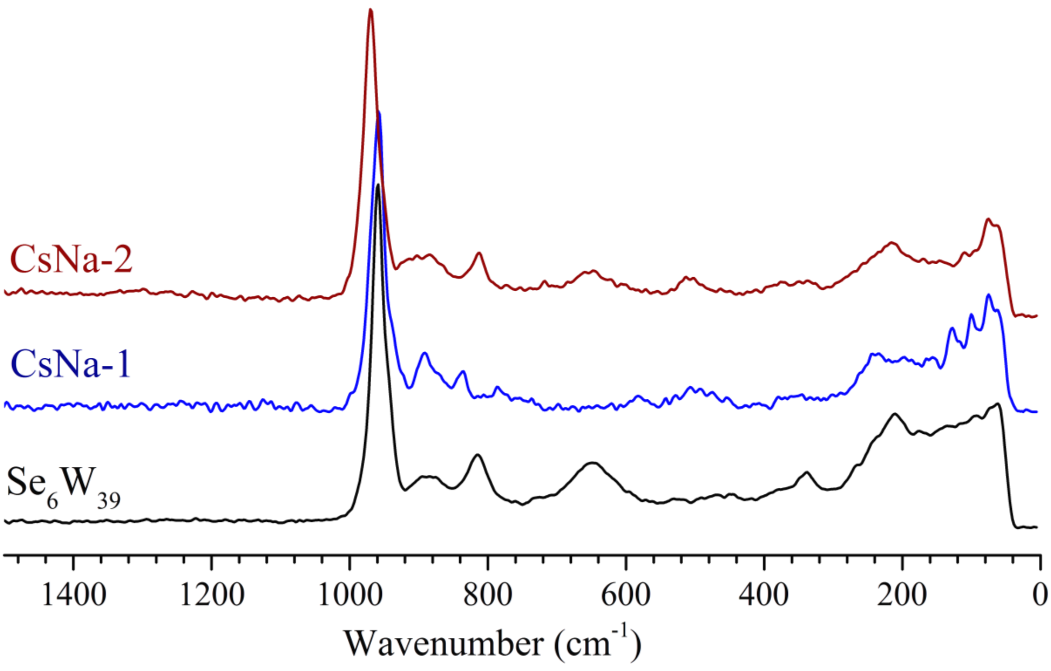

Figure S11. Raman spectra of **CsNa-1** (blue) and **CsNa-2** (brown) in comparison with that of the $Na_{24}[H_6Se_6W_{39}O_{144}]\cdot 74H_2O$ precursor (black).

VII. NMR SPECTROSCOPY MEASUREMENTS

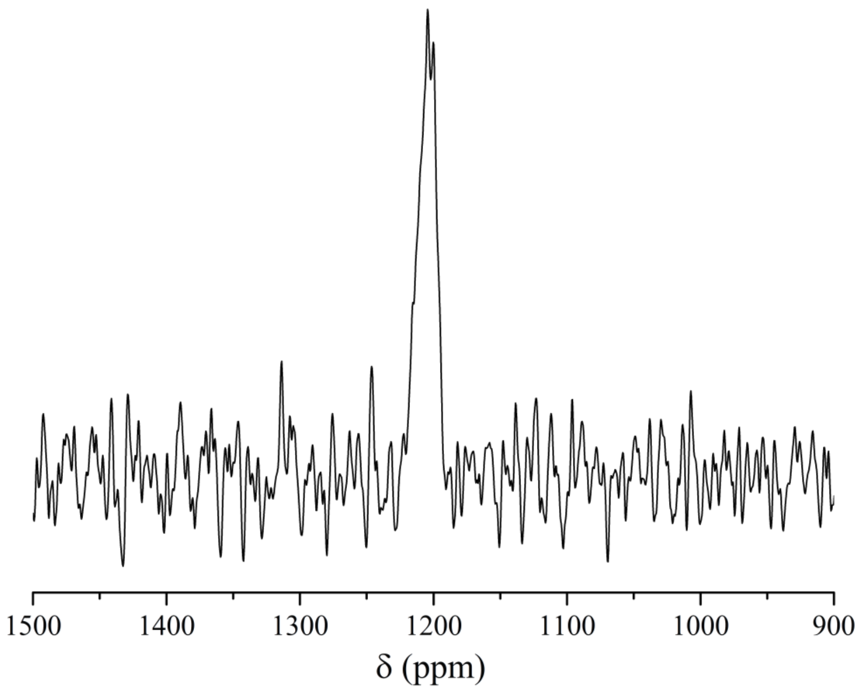

Figure S12. Solid-state ^{77}Se MAS NMR spectrum of **CsNa-1**.

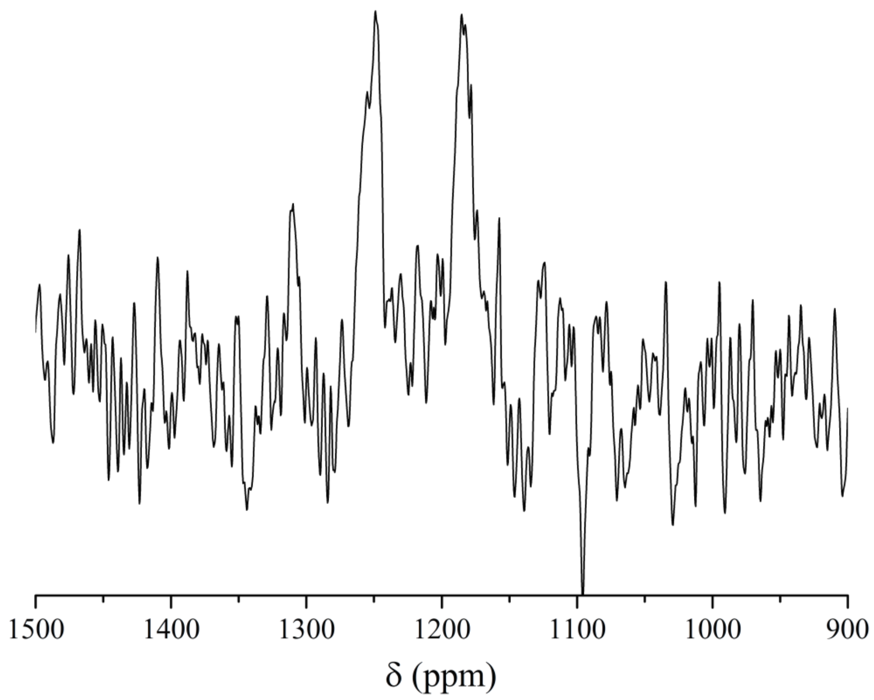

Figure S13. Solid-state ^{77}Se MAS NMR spectrum of **CsNa-2**.

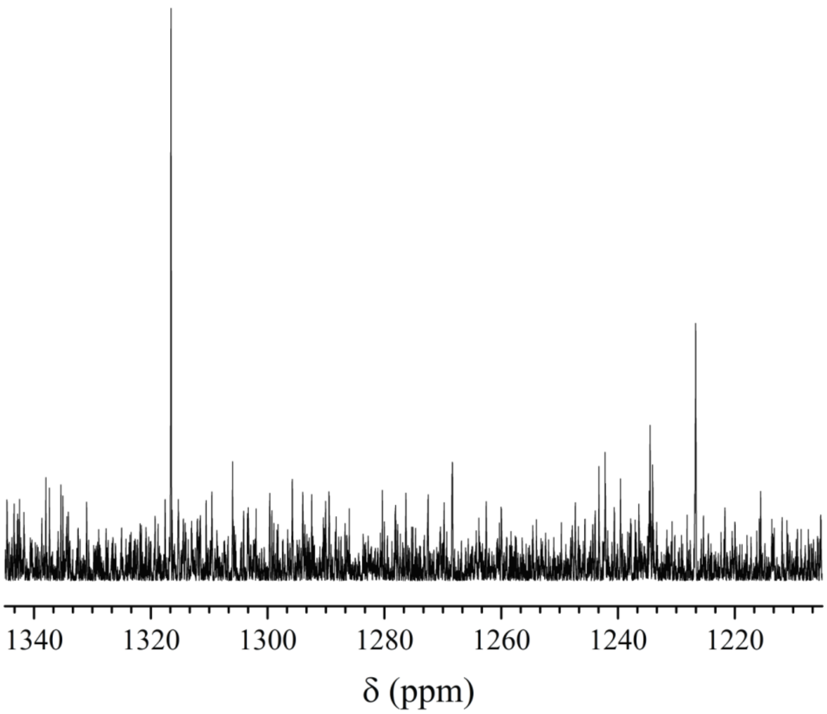

Figure S14. The room temperature ^{77}Se NMR spectrum of **CsNa-2** dissolved in 0.25 M $LiCH_3COO$ solution in H_2O/D_2O (pH 6.2).

VIII. UV-Vis SPECTROSCOPY MEASUREMENTS

The UV-vis spectrum (Fig. S13) of **CsNa-1** solution in 0.25 M aqueous acetate medium (pH 6.7) exhibits strong absorption in the UV associated with ligand-to-metal charge transfer bands of the polyoxopalladatotungstate framework with a well-defined maximum at 227 nm (ε = 74450 M^{-1} cm^{-1} and a broad shoulder at 273 nm (ε = 34153 M^{-1} cm^{-1}), followed by a less intense absorption maximum centered at around 414 nm (ε = 1484 M^{-1} cm^{-1}). The stability test in the same medium shows insignificant decrease of the absorption intensity over 24 h (Fig. S16) that most likely arises not from POM decomposition (taking into account solution ^{77}Se NMR and ESI-MS results) but from the slow precipitation of small solid particles of **CsNa-1**, which is poorly soluble at the concentration required for the measurement. The absence of absorption saturation issues was proven by the reproducibility of ε for measurements on a range of concentrations.

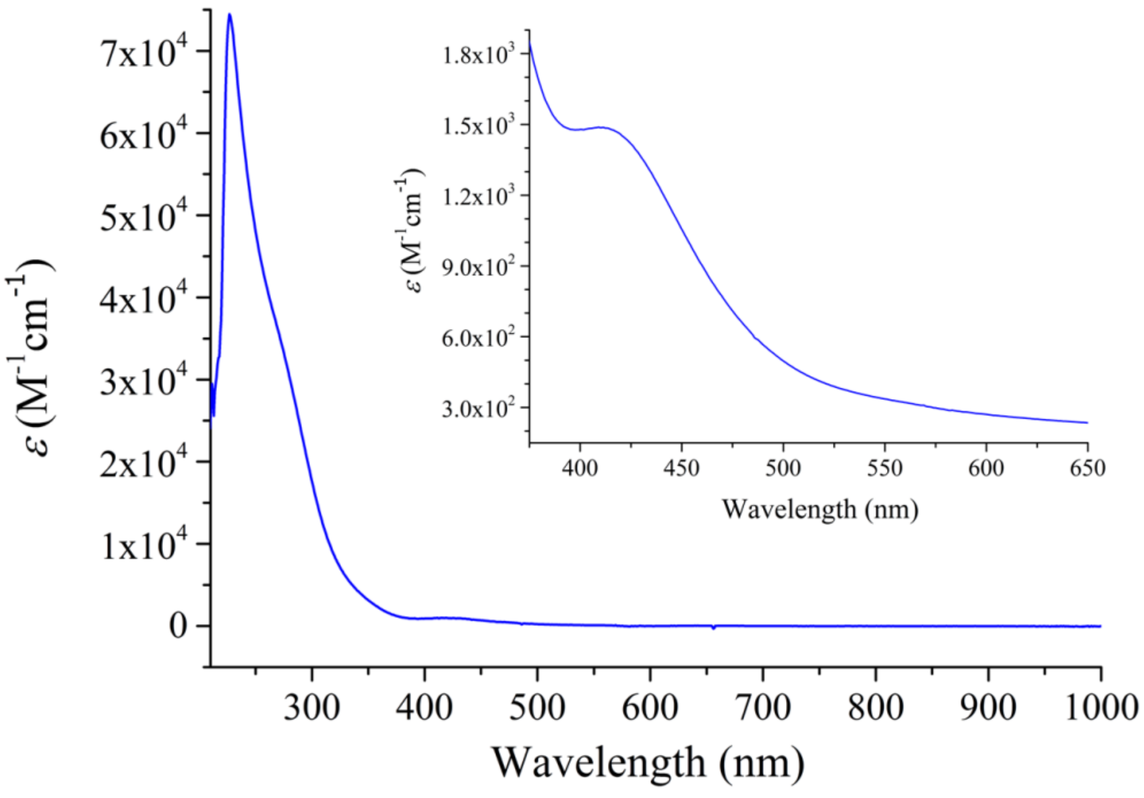

Figure S15. Room-temperature UV-Vis spectrum of **CsNa-1** solution in 0.25 M $NaCH_3COO$ aqueous medium at pH 6.7 (ε values are averaged from the spectra of the solutions with concentrations between 9.1×10^{-6} M and 1.5×10^{-5} M for the UV region and between 2.0×10^{-4} M and 6.0×10^{-4} M for the visible spectral region).

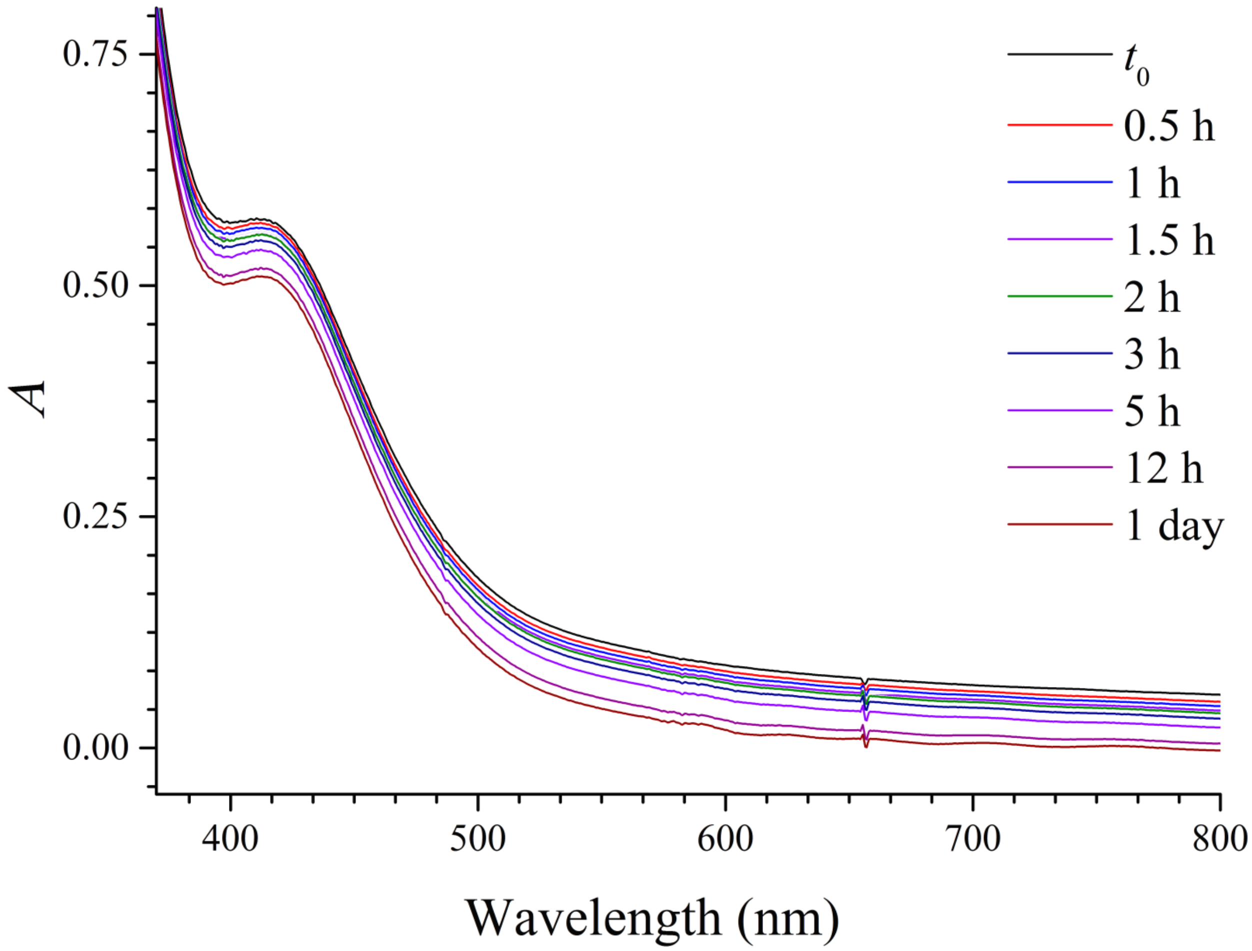

Figure S16. Time-dependent room-temperature UV-Vis spectrum of a 3.0×10^{-4} M **CsNa-1** solution in 0.25 M $NaCH_3COO$ aqueous medium (pH 6.7).

IX. ELECTROSPRAY MASS SPECTROMETRY MEASUREMENTS

Experimental details for the LC-ESI-FTICR-MS analysis. Analyses were performed using a hybrid linear ion trap FTICR mass spectrometer LTQ-FT (Thermo Fisher Scientific, Bremen, Germany) equipped with a 7 T superconducting magnet by infusion. The mass spectrometer was first tuned and calibrated in the negative mode following the standard optimization procedure for all voltages and settings. The complex was dissolved in 80% H_2O and 20% acetone. The transfer capillary temperature was set to 175 °C. Mass spectra were recorded in full scan from 200 to 2000 Da with a resolution of 100.000 at *m*/*z* 400. All data were processed using the Xcalibur software version 2.1.

1. Simulations of the ESI-MS isotope envelopes for the peaks observed in the negative ion mode ESI-MS spectrum of 1 (cmp. Figure 4 in the main text).

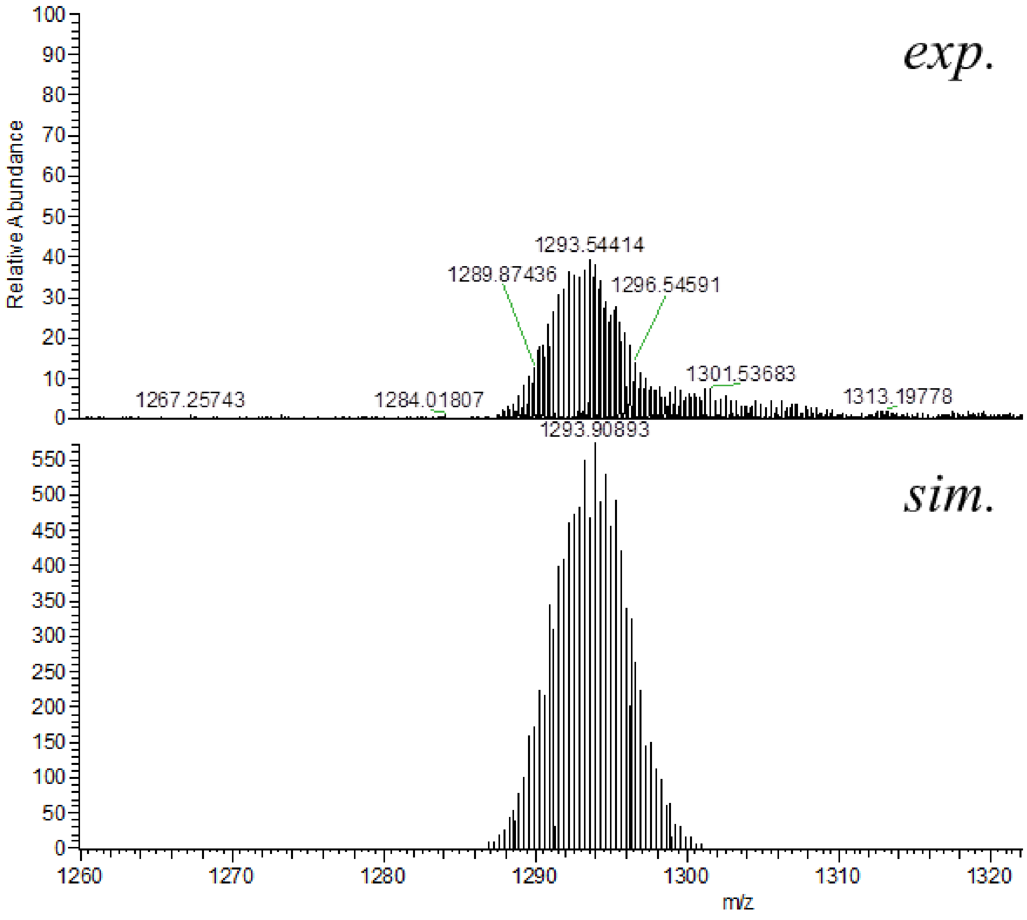

Figure S17. Comparison of the experimental (upper) and calculated (bottom) isotope splitting of the $\{H_9Na_2[Se_2Pd_2W_{14}O_{55}]\}^{3-}$ ion pair (peak I, *see main text*).

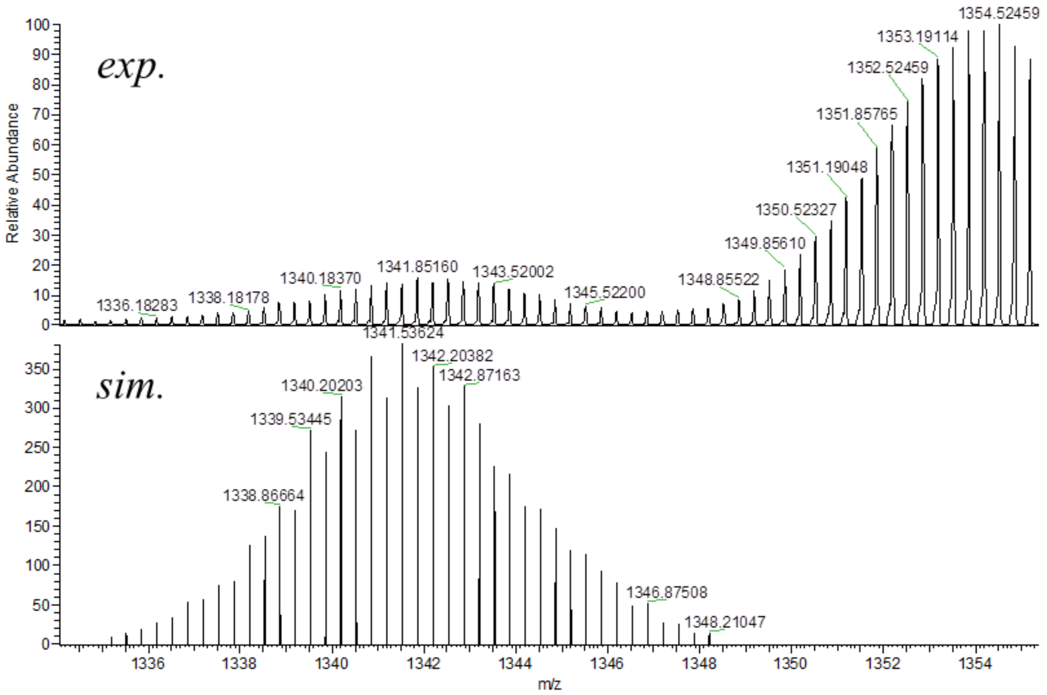

Figure S18. Comparison of the experimental (upper) and calculated (bottom) isotope splitting of the $\{H_8Na_3[Se_2Pd_3W_{14}O_{56}]\}^{3-}$ ion pair (peak II in the main text).

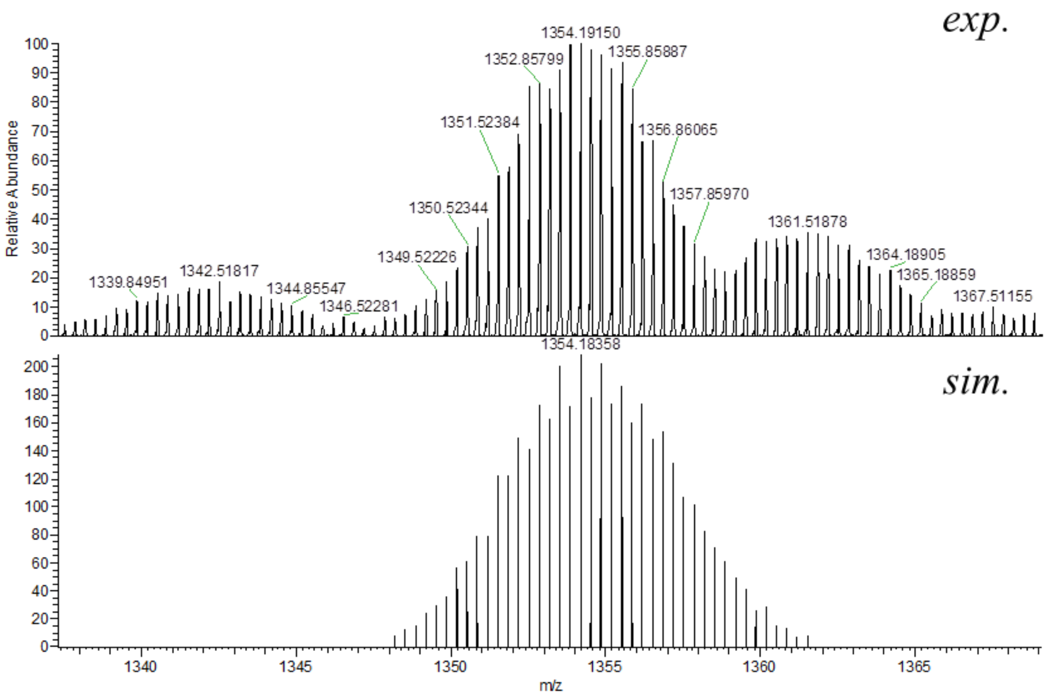

Figure S19. Comparison of the experimental (upper) and calculated (bottom) isotope splitting of the $\{H_8[Se_2Pd_4W_{14}O_{56}H]\}^{3-}$ ion pair (peak III in the main text).

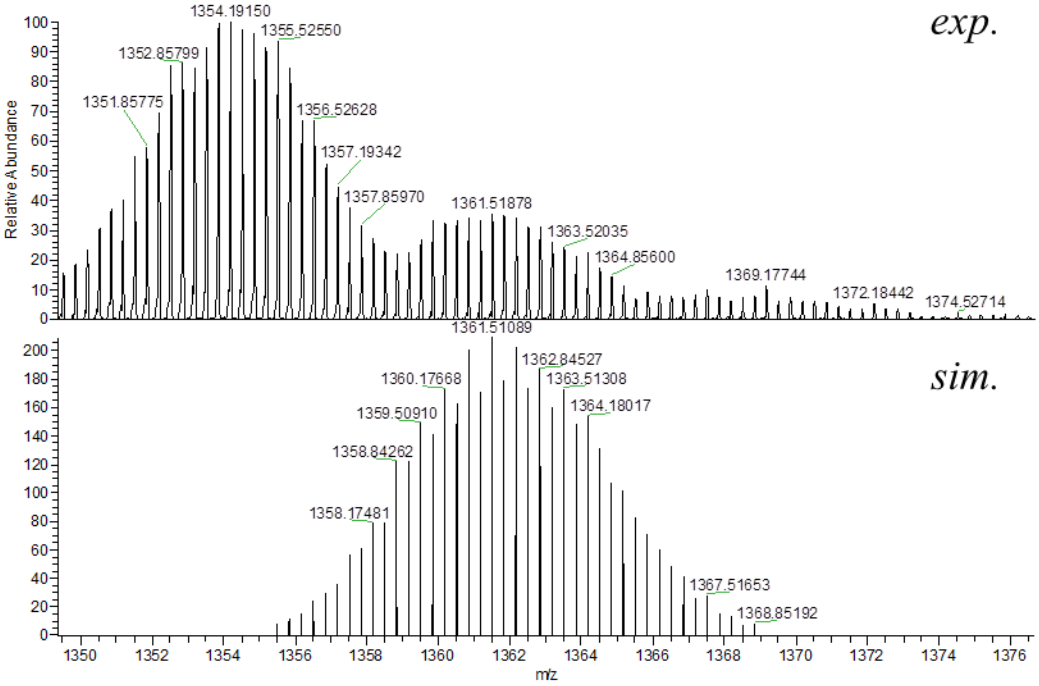

Figure S20. Comparison of the experimental (upper) and calculated (bottom) isotope splitting of the $\{H_7Na[Se_2Pd_4W_{14}O_{56}H]\}^{3-}$ ion pair (peak IV in the main text).

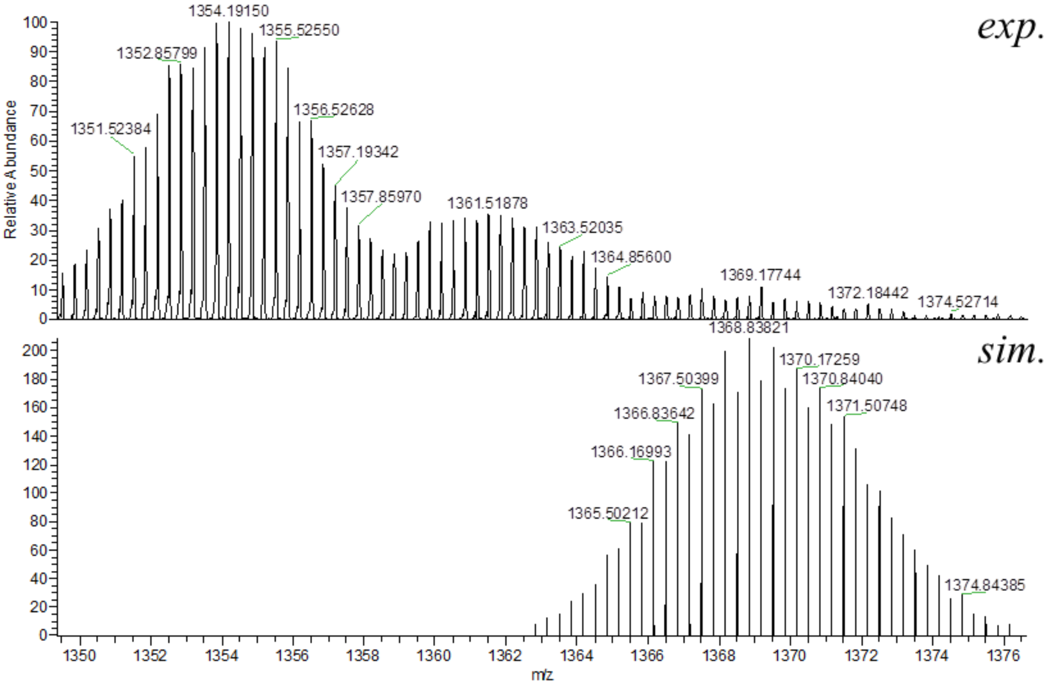

Figure S21. Comparison of the experimental (upper) and calculated (bottom) isotope splitting of the $\{H_6Na_2[Se_2Pd_4W_{14}O_{56}H]\}^{3-}$ ion pair (peak V in the main text).

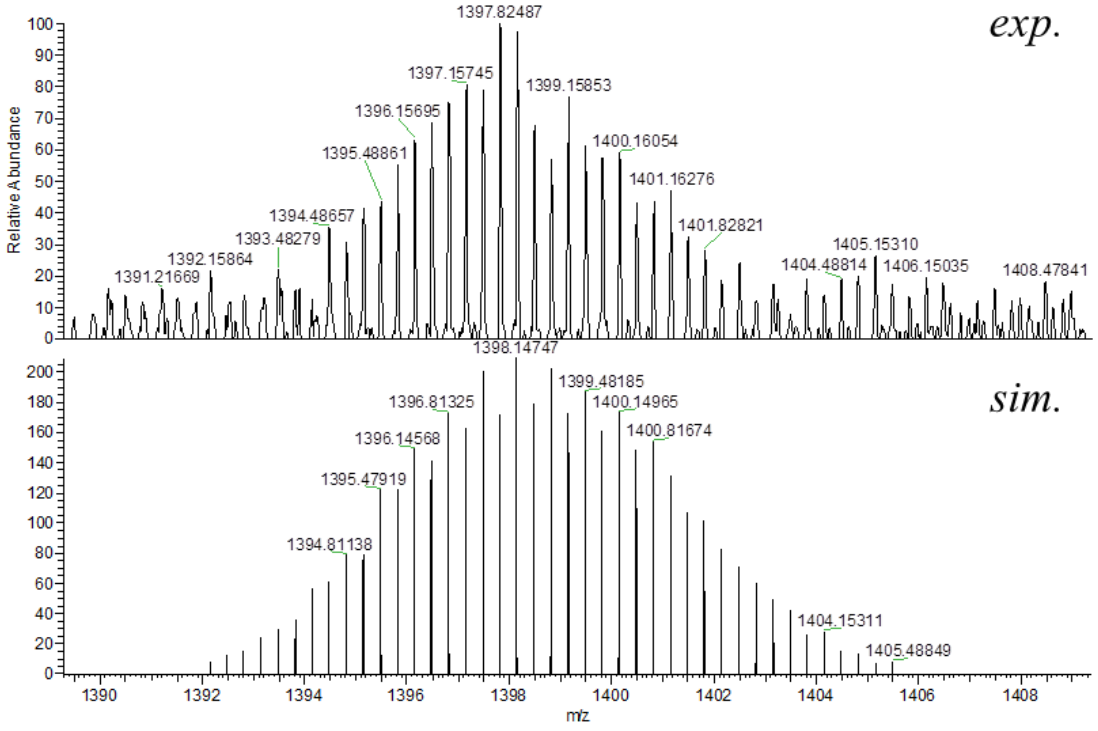

Figure S22. Comparison of the experimental (upper) and calculated (bottom) isotope splitting of the $\{H_2Na_6[Se_2Pd_4W_{14}O_{56}H]\}^{3-}$ ion pair (peak VI in the main text).

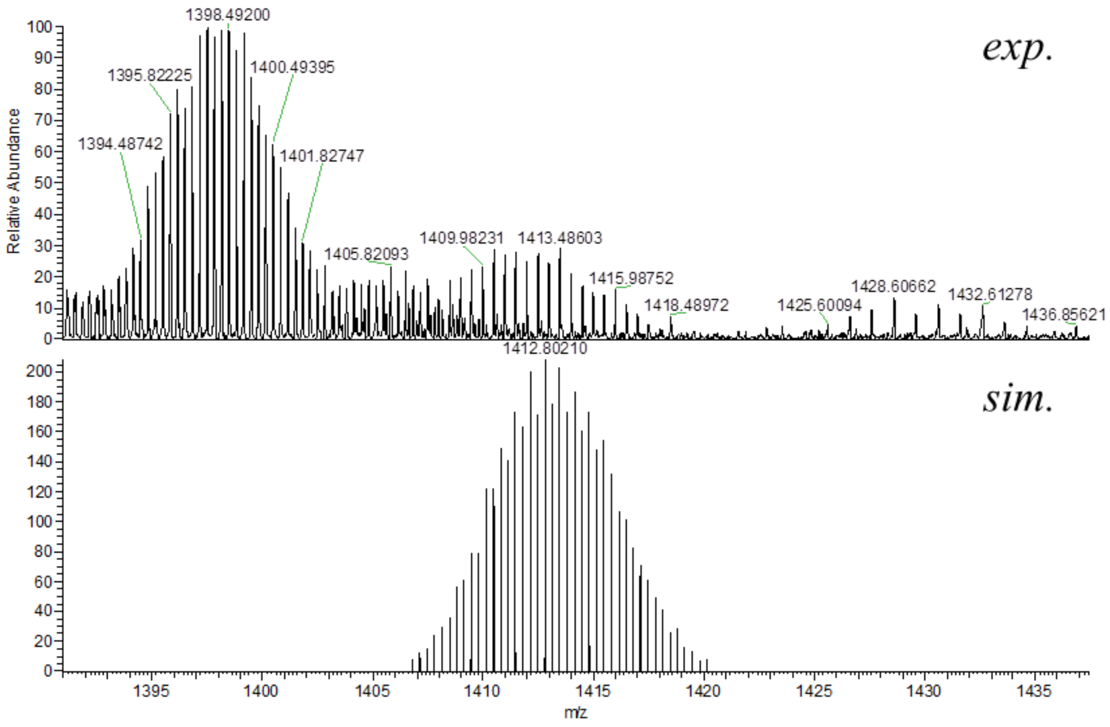

Figure S23. Comparison of the experimental (upper) and calculated (bottom) isotope splitting of the $\{Na_8[Se_2Pd_4W_{14}O_{56}H]\}^{3-}$ ion pair (peak VII in the main text).

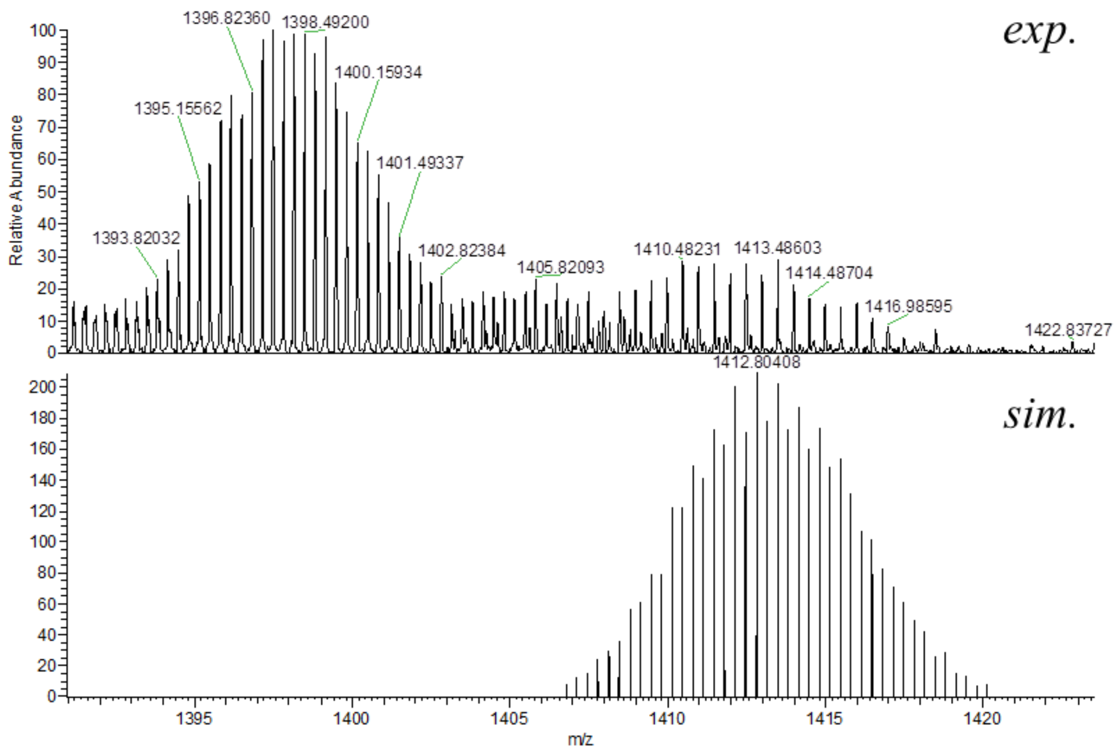

Figure S24. Comparison of the experimental (upper) and calculated (bottom) isotope splitting of the $\{CsH_5Na_2[Se_2Pd_4W_{14}O_{56}H]\}^{3-}$ ion pair (peak VII in the main text).

2. ESI mass spectrum of 2 and simulations for the selected peaks

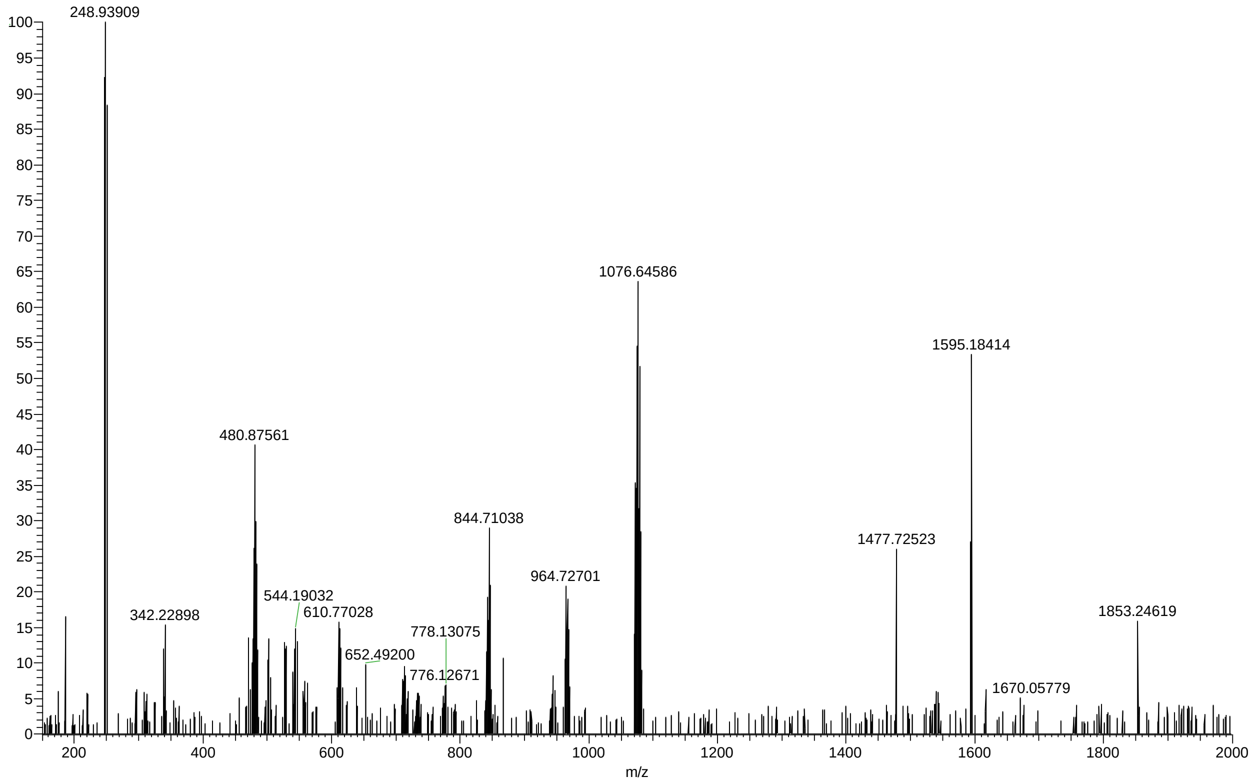

Figure S25. ESI mass spectrum of **2** in H_2O/acetone (80:20 %) solution in negative ion mode.

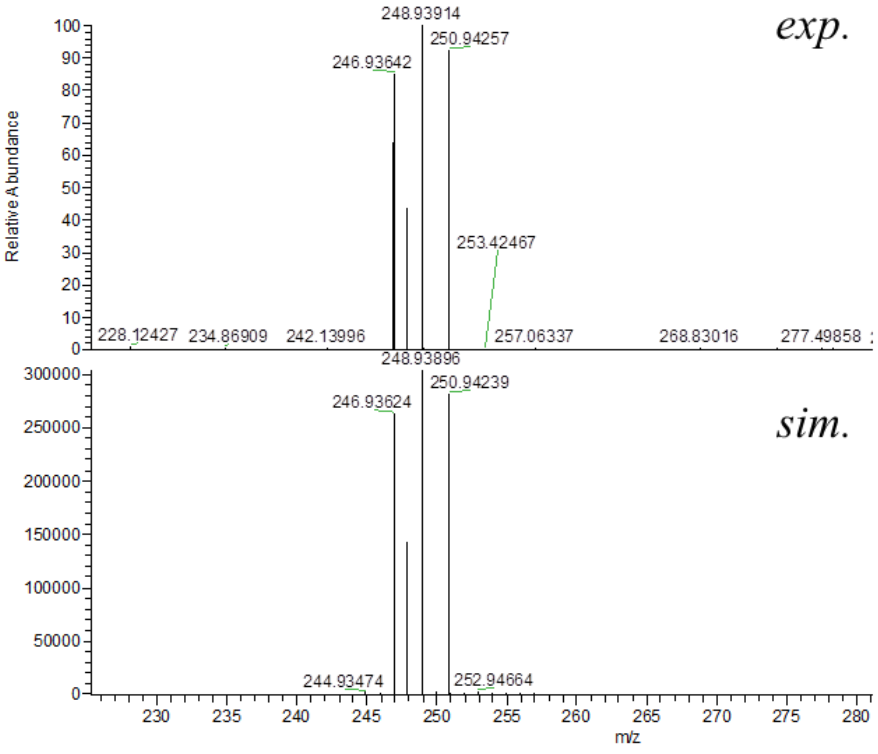

Figure S26. Comparison of the experimental (upper) and calculated (bottom) isotope splitting of the $\{H[WO_4]\}^-$ ion pair.

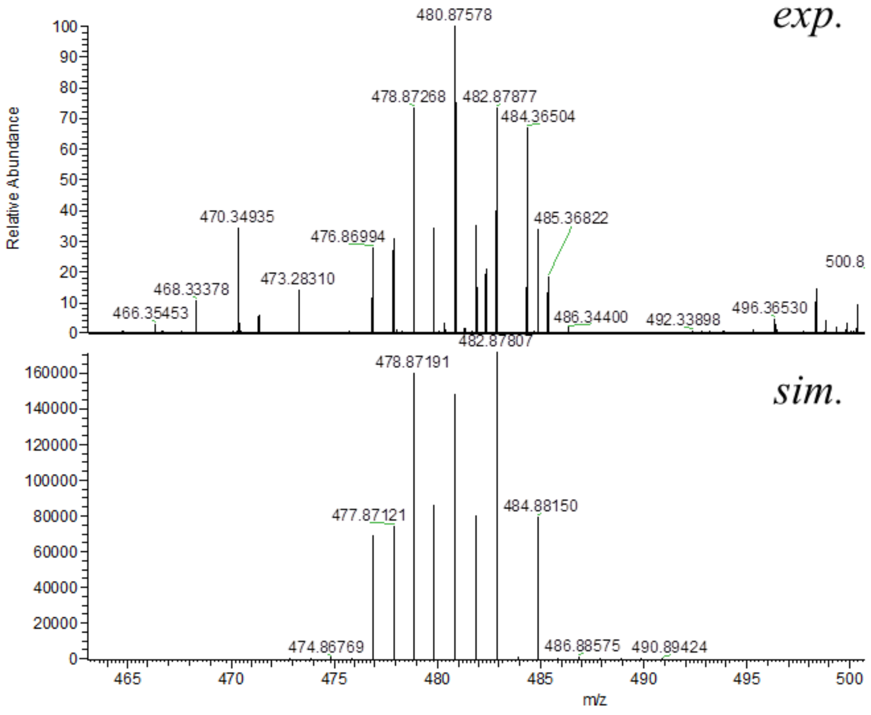

Figure S27. Comparison of the experimental (upper) and calculated (bottom) isotope splitting of the $\{H[W_2O_7]\}^-$ ion pair.

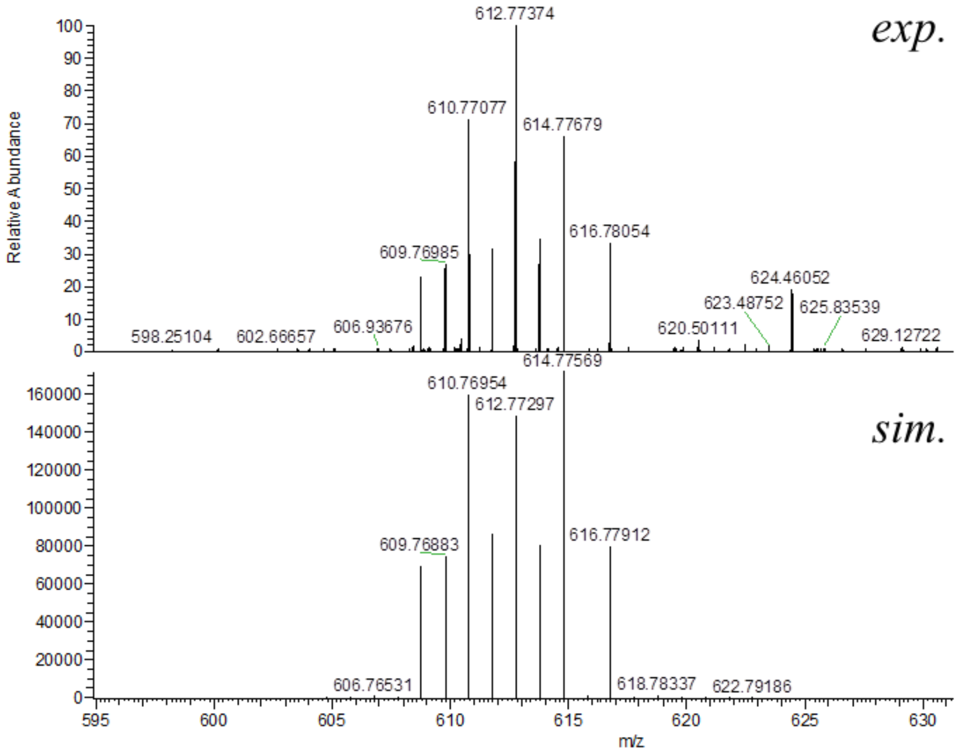

Figure S28. Comparison of the experimental (upper) and calculated (bottom) isotope splitting of the $\{Cs[W_2O_7]\}^-$ ion pair.

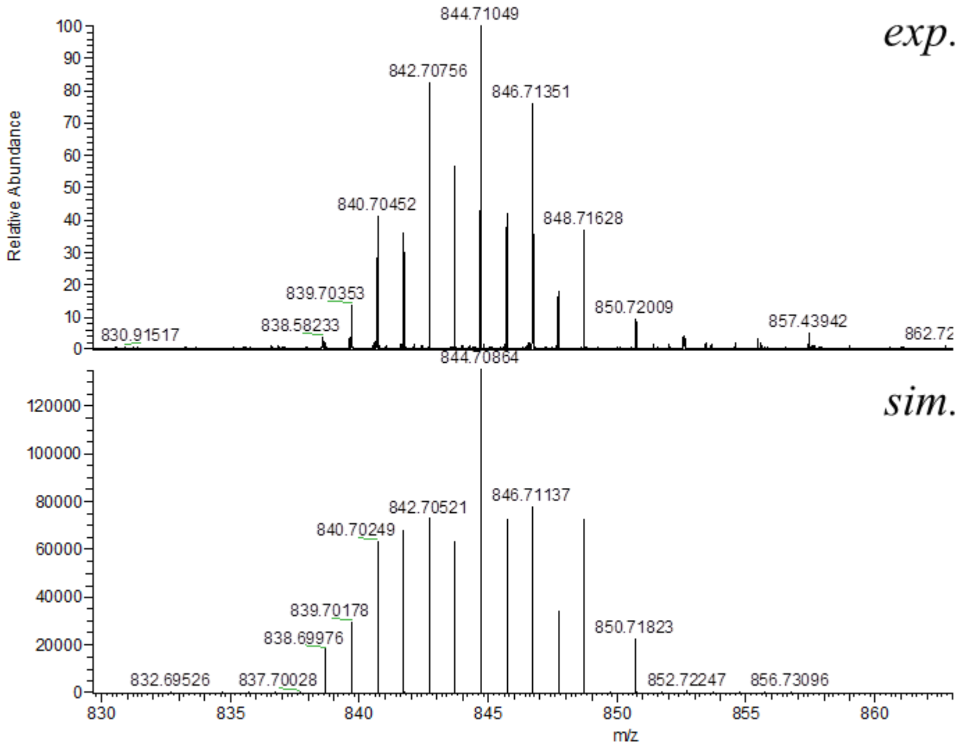

Figure S29. Comparison of the experimental (upper) and calculated (bottom) isotope splitting of the $\{Cs[W_3O_{10}]\}^-$ ion pair.

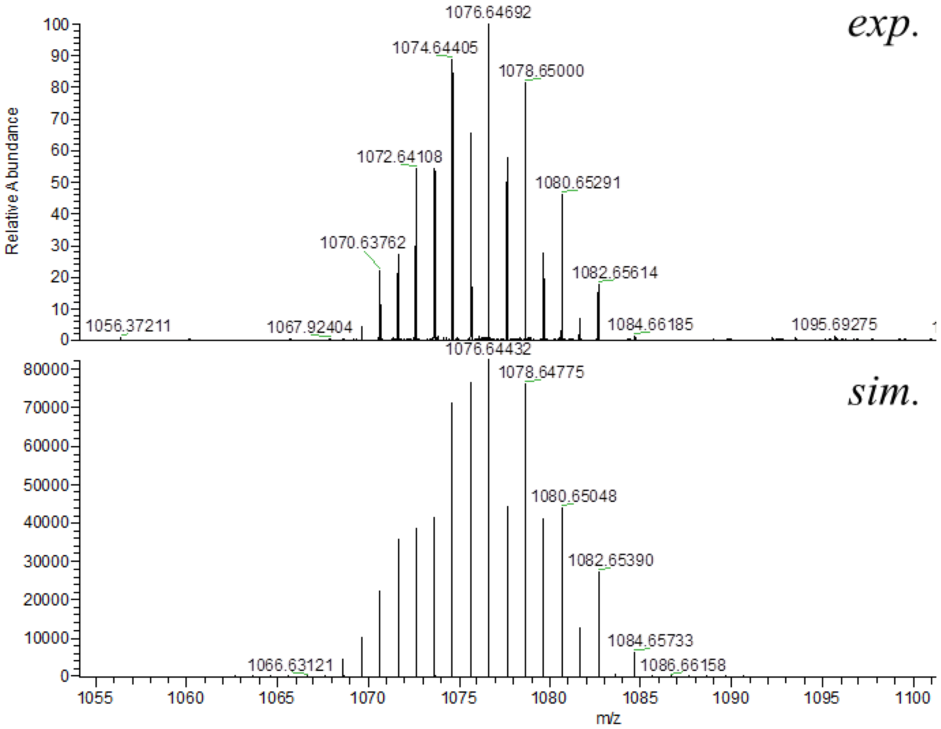

Figure S30. Comparison of the experimental (upper) and calculated (bottom) isotope splitting of the $\{Cs[W_4O_{13}]\}^{-}$ ion pair.

X. REFERENCES

1. I. V. Kalinina, E. V. Peresypkina, N. V. Izarova, F. M. Nkala, U. Kortz, N. B. Kompankov, N. K. Moroz, M. N. Sokolov, *Inorg. Chem*. **2014**, *53*, 2076–2082

2. CrysAlisPro, Agilent Technologies, 1.171.36.28 (release 01-02-2013 CrysAlis171 .NET).

3. G. M. Sheldrick, *Acta Cryst.* **2008**, *A64*, 112–122.

4. (a) B. Godin, J. Vaissermann, P. Herson, L. Ruhlmann, M. Verdaguer, P. Gouzerh, *Chem. Commun*. **2005**, 5624–5626; (b) S. Yao, Z. Zhang, Y. Li, E.-B. Wang, *Dalton Trans*. **2009**, 1786–1791.

5. B. Dawson, *Acta Crystallogr*. **1953**, *6*, 113–126.

6. (a) I. D. Brown and D. Altermatt, *Acta Crystallogr*. **1985**, *B41*, 244–247; (b) K. Knížek, Kalvados – Software for crystal structure and powder diffraction; see http://www.fzu.cz/~knizek/kalvados/index.html
